# Production and Excretion of Polyamines To Tolerate High Ammonia, a Case Study on Soil Ammonia-Oxidizing Archaeon “*Candidatus* Nitrosocosmicus agrestis”

**DOI:** 10.1128/mSystems.01003-20

**Published:** 2021-02-16

**Authors:** Liangting Liu, Mengfan Liu, Yiming Jiang, Weitie Lin, Jianfei Luo

**Affiliations:** a School of Biology and Biological Engineering, South China University of Technology, Guangzhou, People’s Republic of China; b Guangdong Key Laboratory of Fermentation and Enzyme Engineering, South China University of Technology, Guangzhou, People’s Republic of China; c MOE Joint International Research Laboratory of Synthetic Biology and Medicine, South China University of Technology, Guangzhou, People’s Republic of China; Lawrence Berkeley National Laboratory; University of Vienna

**Keywords:** ammonia tolerance, ammonia-oxidizing archaea, *Nitrosocosmicus*, polyamines

## Abstract

Ammonia tolerance is a universal characteristic among the ammonia-oxidizing bacteria (AOB); in contrast, the known species of ammonia-oxidizing archaea (AOA) have been regarded as ammonia sensitive, until the identification of the genus “*Candidatus* Nitrosocosmicus.” However, the mechanism of its ammonia tolerance has not been reported. In this study, the AOA species “*Candidatus* Nitrosocosmicus agrestis,” obtained from agricultural soil, was determined to be able to tolerate high concentrations of NH_3_ (>1,500 μM). In the genome of this strain, which was recovered from metagenomic data, a full set of genes for the pathways of polysaccharide metabolism, urea hydrolysis, arginine synthesis, and polyamine synthesis was identified. Among them, the genes encoding cytoplasmic carbonic anhydrase (CA) and a potential polyamine transporter (drug/metabolite exporter [DME]) were found to be unique to the genus “*Ca.* Nitrosocosmicus.” When “*Ca.* Nitrosocosmicus agrestis” was grown with high levels of ammonia, the genes that participate in CO_2_/HCO_3_^−^ conversion, glutamate/glutamine syntheses, arginine synthesis, polyamine synthesis, and polyamine excretion were significantly upregulated, and the polyamines, including putrescine and spermidine, had significant levels of production. Based on genome analysis, gene expression quantification, and polyamine determination, we propose that the production and excretion of polyamines is probably one of the reasons for the ammonia tolerance of “*Ca.* Nitrosocosmicus agrestis,” and even of the genus “*Ca.* Nitrosocosmicus.”

**IMPORTANCE** Ammonia tolerance of AOA is usually much lower than that of the AOB, which makes the AOB rather than AOA a predominant ammonia oxidizer in agricultural soils, contributing to global N_2_O emission. Recently, some AOA species from the genus “*Ca.* Nitrosocosmicus” were also found to have high ammonia tolerance. However, the reported mechanism for the ammonia tolerance is very rare and indeterminate for AOB and for AOA species. In this study, an ammonia-tolerant AOA strain of the species “*Ca.* Nitrosocosmicus agrestis” was identified and its potential mechanisms for ammonia tolerance were explored. This study will be of benefit for determining more of the ecological role of AOA in agricultural soils or other environments.

## INTRODUCTION

The ammonia-oxidizing archaea (AOA), composed of *Nitrosopumilales*, “*Candidatus* Nitrosotaleales,” “*Candidatus* Nitrosocaldales,” and *Nitrososphaerales* within the phylum *Thaumarchaeota*, play a major role in the global nitrogen cycle by mediating the conversion of ammonia to nitrite (1–3). AOA are ubiquitous and estimated to represent 1 to 5% of all prokaryotes in soils (1, 4). The organisms *Nitrososphaera* and “*Candidatus* Nitrosocosmicus” in the order *Nitrososphaerales* are regarded as the main AOA groups distributed in terrestrial soil environments. Until now, four strains belonging to *Nitrososphaera* and five strains belonging to “*Ca.* Nitrosocosmicus” have been reported (5, 6). Although it is a sister cluster of *Nitrososphaera*, “*Ca.* Nitrosocosmicus” has many properties different from those of *Nitrososphaera* and other AOA clusters, among them the ability to tolerate high concentration of ammonia (7–9).

Because the ammonia affinity of AOA is dozens or hundreds of times higher than that of the ammonia-oxidizing bacteria (AOB) (10–12), members of the AOA have usually been observed to have higher abundance than AOB in habitats with low ammonia, such as the oligotrophic oceans (13, 14). Generally, AOA species have been reported to have low tolerance to un-ionized ammonia (1 to 800 μM) in contrast to the AOB species (up to 74 mM) (Table 1). In agricultural soils, overfertilization results in high concentration of ammonia, which contributes to the activation of AOB and the limitation of AOA, even though the AOA groups sometimes dominate in numbers (15, 16). Due to the high ammonia, the nitrifier denitrification activity mediated by AOB is promoted while the activity of AOA is suppressed, which makes the AOB groups the main producers of nitrous oxide (N_2_O), contributing to global N_2_O emission from agricultural soils (17–19).

**TABLE 1:** Ammonia and pH tolerances of AOA strains

Organism	Strain	Source	Inhibitory NH_4_^+^ (mM)^*a*^/NH_3_ (μM)^*b*^	Temp (°C)	pH^*c*^	Reference
“*Ca.* Nitrosopumilales”	Nitrosopumilus maritimus SCM1	Tropical marine aquarium	2 (8.2)/225.65	30	6.8–8.2	11
	“*Ca.* Nitrosopumilus koreensis AR1”	Marine sediment	4 (8.2)/329.68	25	NA	80
	“*Ca.* Nitrosoarchaeum koreensis” MY1	Agricultural soil	20 (7.0)/112.71	25	6.0–8.0	30
	“*Ca.* Nitrosotenuis chungbukensis” MY2	Agricultural soil	20 (7.0)/112.71	25	6.5–8.5	81
	“*Ca.* Nitrosotenuis cloacae” SAT1	Wastewater treatment plant	5 (6.5)/11.82	29	5.0–7.0	28
	Nitrosopumilus cobalaminigenes HCA1	50 m depth marine water	10 (7.3)/78.31	20	6.8–8.1	82
	Nitrosopumilus ureiphilus PS0	Marine surface sediment	20 (6.8)/49.79	20	5.9–8.1	82
	Nitrosopumilus oxyclinae HCE1	17 m depth marine water	1 (7.3)/7.83	20	6.4–7.8	82
	“*Ca.* Nitrosotenuis aquarius” AQ6f	Freshwater aquarium biofilter	3 (8.5)/607.18	30	NA	27

“*Ca.* Nitrosotaleales”	“*Ca.* Nitrosotalea devanaterra” Nd1	Acid soil	50 (4.5)/1.11	28	4.0–5.5	83

*Nitrososphaerales*	“*Ca.* Nitrososphaera gargensis” Ga9.2	Hot spring	3 (7.4)/166.09	46	NA	84
	Nitrososphaera viennensis EN76	Garden soil	20 (7.5)/778.81	37	6.0–8.5	85
	“*Ca.* Nitrososphaera” sp. strain JG1	Agricultural soil	20 (6.5)/80.71	37	6.0–8.0	34
	“*Ca.* Nitrosocosmicus oleophilus” MY3	Coal tar-contaminated sediment	100 (7.0)/796.04	30	5.5–8.5	9
	“*Ca.* Nitrosocosmicus franklandus” C13	Arable soil	100 (7.5)/3894.07	37	6–8.5	8
	“*Ca.* Nitrosocosmicus exaquare” G61	Wastewater treatment plant	20 (8.0)/1485.64	30	NA	7
	“*Ca.* Nitrososphaera” sp. OTU8	Wastewater treatment plant	3 (7.5)/116.82	37	7.0–8.0	86
	“*Ca.* Nitrosocosmicus agrestis” SS	Vegetable soil	200 (7.0)/1592.07	30	5.5–8.0	This study

AOB	Nitrosomonas europaea	Sewage disposal plants	400 (8.0)/29712.72	30		24
	Nitrosomonas ureae	Oligotrophic freshwater and natural soils	200 (8.0)/14856.36	30		24
	Nitrosomonas marina	Marine environments	200 (8.0)/14856.36	30		24
	Nitrosolobus multiformis	Soils (not acid)	50 (8.0)/3714.09	30		24
	Nitrosovibrio tenuis	Soils, rocks and freshwater	100 (8.0)/7428.18	30		24
	Nitrosospira briensis	Soils, rocks and freshwater	200 (8.0)/14856.36	30		24
	Nitrosococcus oceani	Marine environments	1,000 (8.0)/74281.79	30		24

a Inhibitory NH_4_^+^ (mM), ammonium chloride concentration that completely or almost inhibited AOA ammonia oxidization. The numbers in parentheses are the pH of the experiments.

b NH_3_ (μM), free ammonia concentration, calculated according to total ammonia, pH, and temperature, estimating by formulas published by Emerson et al. (64).

c The range of pH at which the organism can grow.

It seems that the AOB predominate as the active nitrifiers that are responsible for the ammonia oxidation in agricultural soils. However, the AOA groups have been observed to have ammonia oxidation activity and to make a small contribution (10 to 20%) to N_2_O production (17–19); they are not always inhibited by the application of large amount of nitrogen fertilizers (20–23). In particular, some strains from the genus “*Ca.* Nitrosocosmicus” obtained from soil, sediment, and wastewater treatment plants were observed to tolerate more than 1,000 μM un-ionized ammonia (7, 8) (Table 1). These findings suggested that some species of “*Ca.* Nitrosocosmicus” and even some unknown AOA species were probably able to tolerate high ammonia levels in agricultural soils.

Ammonia tolerance is well-known among AOB, especial for the species of *Nitrosococcus* (24). However, the mechanism for high-ammonia tolerance has been rarely reported for the AOB species, not to mention for the AOA species. In this study, an AOA strain named “*Candidatus* Nitrosocosmicus agrestis” SS that previously obtained from agricultural soil was observed to be able to tolerate 1,592 μM un-ionized ammonia (Table 1). Based on comparative genomic and transcriptional expression analyses, as well as metabolite determination, a potential mechanism for “*Ca.* Nitrosocosmicus agrestis” SS tolerance of high ammonia concentrations was proposed. This study identified some new features to more fully characterize the “*Ca.* Nitrosocosmicus” clade and uncovered some novel biochemical, physiological, and ecological roles of AOA in the global environment.

## RESULTS

### New strain of “*Ca.* Nitrosocosmicus” from agricultural soil.

**FIG 1:**
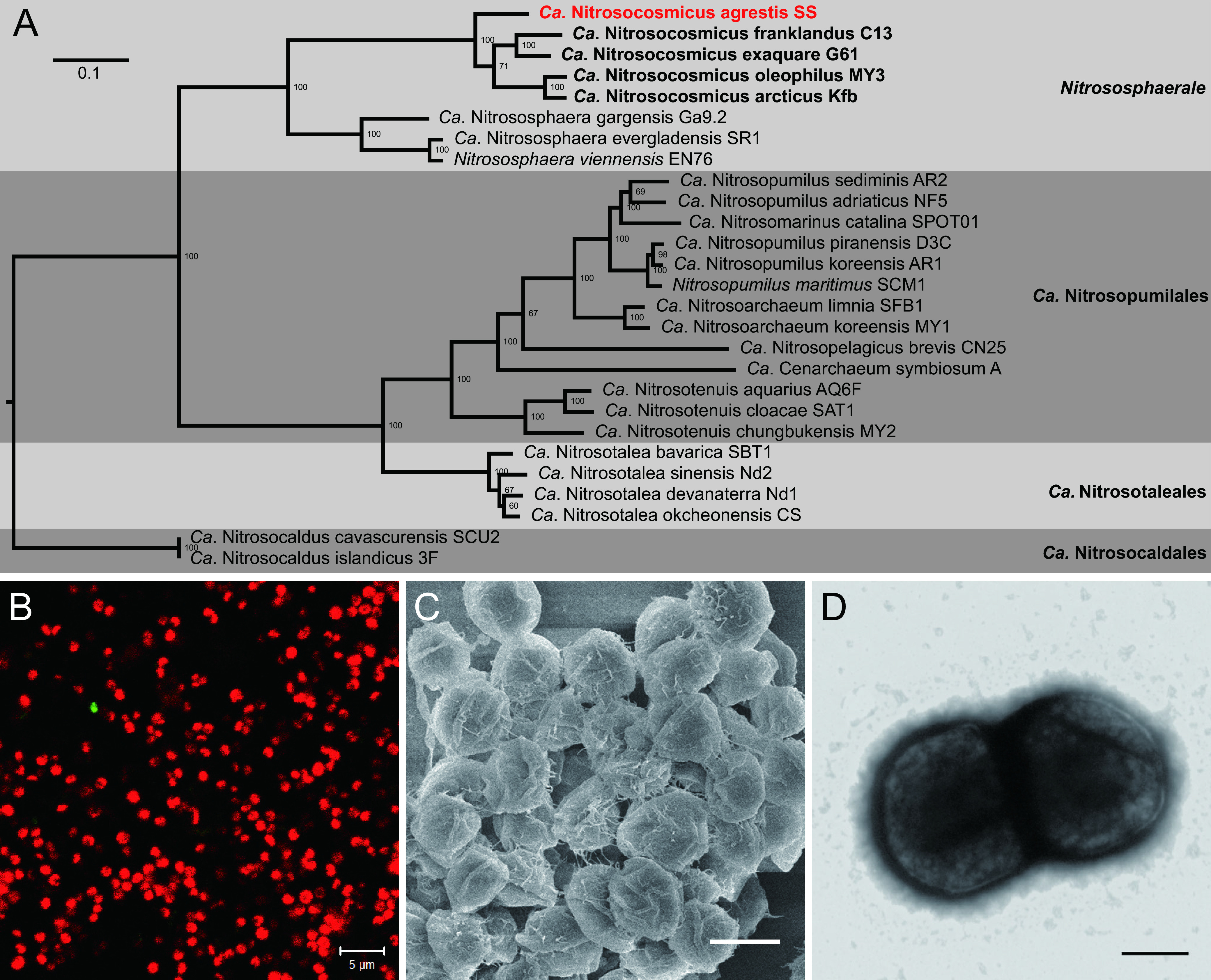
Identification of “*Candidatus* Nitrosocosmicus agrestis.” (A)The tree was inferred on 43 concatenated universal marker proteins from the *Thaumarchaeota* by maximum likelihood with IQ-TREE using an LG+F+R4 model and an ultrafast bootstrap value of 1,000. (B) FISH observation of “*Ca.* Nitrosocosmicus agrestis” cells. Bar = 5 μm. Red, Alexa Fluor 546 labeled archaeal 16S rRNA; green, Alexa Fluor 488-labeled bacterial 16S rRNA. (C) SEM observation of “*Ca.* Nitrosocosmicus agrestis” aggregates. Bar = 1 μm. (D) SEM observation of “*Ca.* Nitrosocosmicus agrestis” aggregates. Bar = 200 nm.

Phylogenomic analysis based on 43 concatenated universal marker protein sequences indicated that the AOA strain SS was closely related to the genus “*Ca.* Nitrosocosmicus” and forms a deep branch in the clade “*Ca.* Nitrosocosmicus” (Fig. 1A). The average nucleotide identity (ANI) of strain SS to the closely related strains was 72.03% to 72.81%, and the average amino acid identity (AAI) was 71.16% to 72.68% (see Fig. S1 in the supplemental material); the ANI and AAI values are above the proposed genus and below the proposed species boundary thresholds (25, 26). In accordance with the phylogenomic and genomic ANI/AAI analyses, strain SS was assigned to the genus “*Ca.* Nitrosocosmicus” and referred to as “*Ca.* Nitrosocosmicus agrestis.” The cells of “*Ca.* Nitrosocosmicus agrestis” are irregular spheres, 0.8 to 1.2 μm in diameter, and appeared in pairs or aggregates embedded in the extracellular matrix (Fig. 1B to D).

10.1128/mSystems.01003-20.1 FIG S1 Heat maps showing pairwise average nucleotide identity (ANI) and average amino acid identity (AAI) values inferred from the available AOA genomes. The ANI and AAI were calculated using JSpeciesWS and compareM (https://github.com/dparks1134/CompareM), respectively. Download FIG S1, PDF file, 0.4 MB. Copyright © 2021 Liu et al. 2021 Liu et al. https://creativecommons.org/licenses/by/4.0/ This content is distributed under the terms of the Creative Commons Attribution 4.0 International license.

**FIG 2:**
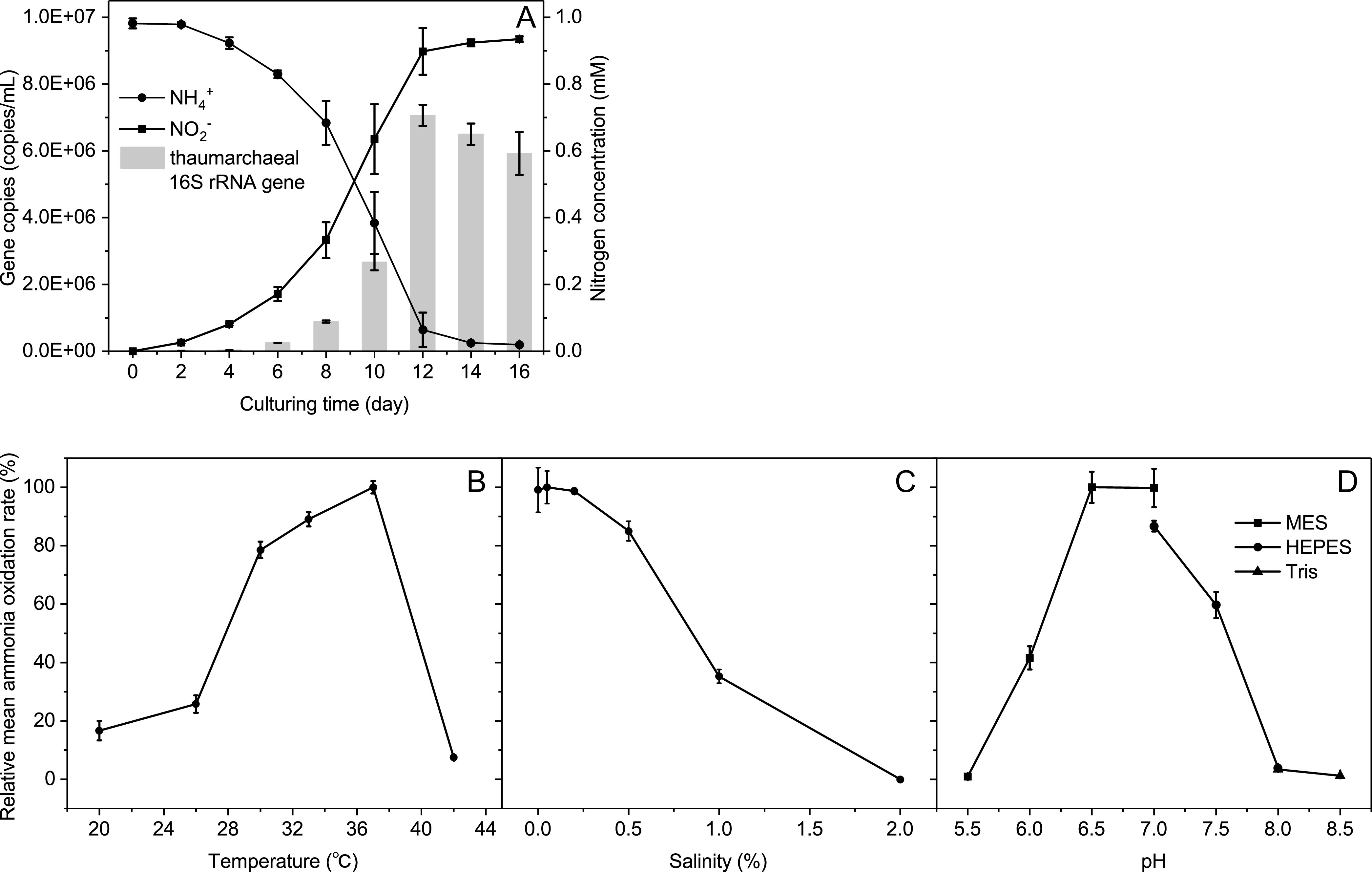
Basic physiological characteristics of “*Ca.* Nitrosocosmicus agrestis” cells. (A) Growth curve. (B) Influence of temperature on the ammonia oxidation activity. (C) Influence of salinity on the ammonia oxidation activity. (D) Influence of pH on the ammonia oxidation activity. The relative ammonia oxidation rate was calculated based on the nitrite concentration on the eighth day. All error bars indicate the standard errors of the means for biological triplicates.

The calculation of nitrite production over time (Fig. 2A) suggested that the generation time of “*Ca.* Nitrosocosmicus agrestis” was 30.2 h (under 30°C), which is shorter than that of “*Candidatus* Nitrosocosmicus exaquare” G61 (51.7 h), “*Candidatus* Nitrosocosmicus franklandus” C13 (40 h), and “*Candidatus* Nitrosocosmicus oleophilus” MY3 (77.4 h) (7–9). After the oxidation of 1 mM NH_4_^+^, the cell density in culture was determined to be approximately 7.06 × 10^6^ cells ml^−1^, which is similar to that of “*Ca*. Nitrosocosmicus franklandus” C13 (7.6 × 10^6^ cells ml^−1^) and “*Ca*. Nitrosocosmicus oleophilus” MY3 (3.2 × 10^6^ cells ml^−1^) but lower than that of “*Ca*. Nitrosocosmicus exaquare” G61 (5.6 × 10^7^ cells ml^−1^) (7–9).

The ammonia-oxidizing activity of “*Ca.* Nitrosocosmicus agrestis” was observed to be optimal at 37°C and nearly inhibited at 42°C (Fig. 2B); the activity was optimal at 0 to 0.2% NaCl and completely suppressed by 2% NaCl (Fig. 2C). “*Ca.* Nitrosocosmicus agrestis” was able to tolerate NaCl (i.e., it retained ∼35% of activity under 1% NaCl), which is much higher than the levels of many other terrestrial AOA strains, such as “*Candidatus* Nitrosotenuis aquarius” AQ6f (0.1%) (27), “*Candidatus* Nitrosotenuis cloacae” SAT1 (0.03%) (28), “*Candidatus* Nitrosotenuis uzonensis” N4 (0.1%) (29), and “*Candidatus* Nitrosoarchaeum koreensis” MY1 (0.4%) (30). Ammonia-oxidizing activity was observed at pH values ranging from 5.5 to 8.0 and determined to be optimal at pH 6.5 to 7.0 (Fig. 2D).

“*Ca.* Nitrosocosmicus agrestis” was also able to use urea as the sole energy and nitrogen source; the ammonia oxidation rate when urea was the sole energy source was only 1/10 of that when ammonia was the energy source, while the relative expression levels of genes relating to urea transport and hydrolysis were conversely 11 to 13 times higher (Fig. S2). The half-maximal inhibitory concentrations (IC_50_s) of nitrification inhibitors, including allylthiourea (ATU), dicyanodiamide (DCD), and 3,4-dimethylpyrazole phosphate (DMPP), for “*Ca.* Nitrosocosmicus agrestis” were determined to be 445.1, 947.1 and 488.0 μM, respectively (Fig. S3), which are similar to observations for other AOA species (31–33). In contrast, the IC_50_ of nitrapyrin (NP) for “*Ca.* Nitrosocosmicus agrestis” was only 0.599 μM (Fig. S3), which is about 1/100 to 6/100 of that for Nitrososphaera viennensis EN76 (32), “*Candidatus* Nitrososphaera” sp. strain JG1 (34), “*Ca.* Nitrosoarchaeum koreensis” MY1 (30), and “*Candidatus* Nitrosotalea devanaterra” (31).

10.1128/mSystems.01003-20.2 FIG S2 Effect of urea on nitrite (A) and ammonia (B) concentration in the ammonia oxidation process and relative gene expression levels in response to urea (C). 1A, 0.1A, and 0A correspond to 1 mM, 0.1 mM, and 0 mM NH_4_Cl; 1U indicates 1 mM urea. Amt, Amt ammonium transporter; UreC, urease subunit alpha; UT, urea transporter. Error bars indicate standard errors of the means for biological triplicates. Download FIG S2, TIF file, 0.4 MB. Copyright © 2021 Liu et al. 2021 Liu et al. https://creativecommons.org/licenses/by/4.0/ This content is distributed under the terms of the Creative Commons Attribution 4.0 International license.

10.1128/mSystems.01003-20.3 FIG S3 Influences of nitrification inhibitor on the ammonia oxidation activity of “*Ca.* Nitrosocosmicus agrestis.” The relative ammonia oxidation rate was calculated based on the nitrite concentration on the eighth day. All error bars indicate standard errors of the means for biological triplicates. DCD, dicyanodiamide; ATU, allylthiourea; DMPP, 3,4-dimethylpyrazole phosphate; NP, nitrapyrin. Download FIG S3, TIF file, 0.4 MB. Copyright © 2021 Liu et al. 2021 Liu et al. https://creativecommons.org/licenses/by/4.0/ This content is distributed under the terms of the Creative Commons Attribution 4.0 International license.

### Ammonia tolerance of “*Ca.* Nitrosocosmicus agrestis” and the other AOA strains.

**FIG 3:**
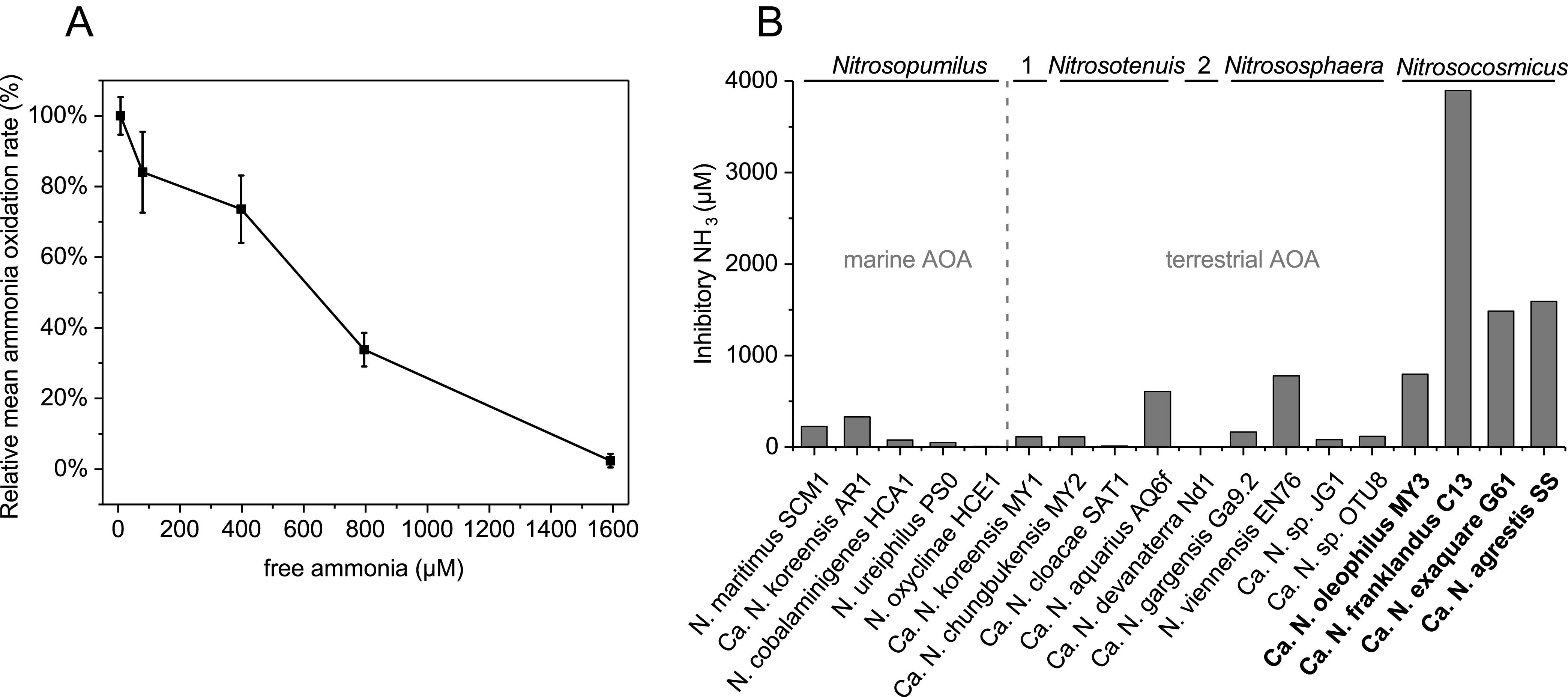
Ammonia tolerance of *Nitrosocosmicus*-like AOA. (A) Influence of the un-ionized ammonia on the ammonia oxidation activity of “*Ca.* Nitrosocosmicus agrestis.” The relative ammonia oxidation rate was calculated based on the nitrite concentration on the eighth day. Error bars indicate standard errors of the means for biological triplicates. (B) Inhibitory FA concentration of different AOA strain. 1, “*Ca.* Nitrosoarchaeum”; 2, “*Ca*. Nitrosotalea.”

The ammonia oxidation activity of “*Ca.* Nitrosocosmicus agrestis” decreased with increases in the initial ammonia concentration; about 75%, 35%, and 5% of the activity were maintained when the concentration of un-ionized ammonia (NH_3_) reached 400, 800, and 1,592 μM, respectively (Fig. 3A). Ammonia-oxidizing strains of “*Ca.* Nitrosocosmicus” were usually reported to have higher tolerance either to NH_3_ or to ionized ammonia (NH_4_^+^) than the other AOA strains. The inhibitory concentration of NH_3_ for strains of the genus “*Ca.* Nitrosocosmicus” was determined to range from 796 to 3,894 μM; except for strains of “*Ca.* Nitrosocosmicus” and the terrestrial strains Nitrososphaera viennensis EN76 and “*Ca.* Nitrosotenuis aquarius” AQ6f, the inhibitory concentrations of NH_3_ for other strains are less than 500 μM (Fig. 3B). In fact, the inhibition by NH_3_ of known AOA varies between strains as well as between genera; only strains of “*Ca.* Nitrosocosmicus” are able to tolerate high ammonia levels, which suggests that ammonia tolerance is probably prevalent among strains of this genus.

### Genomic features of “*Ca.* Nitrosocosmicus agrestis.”

**FIG 4:**
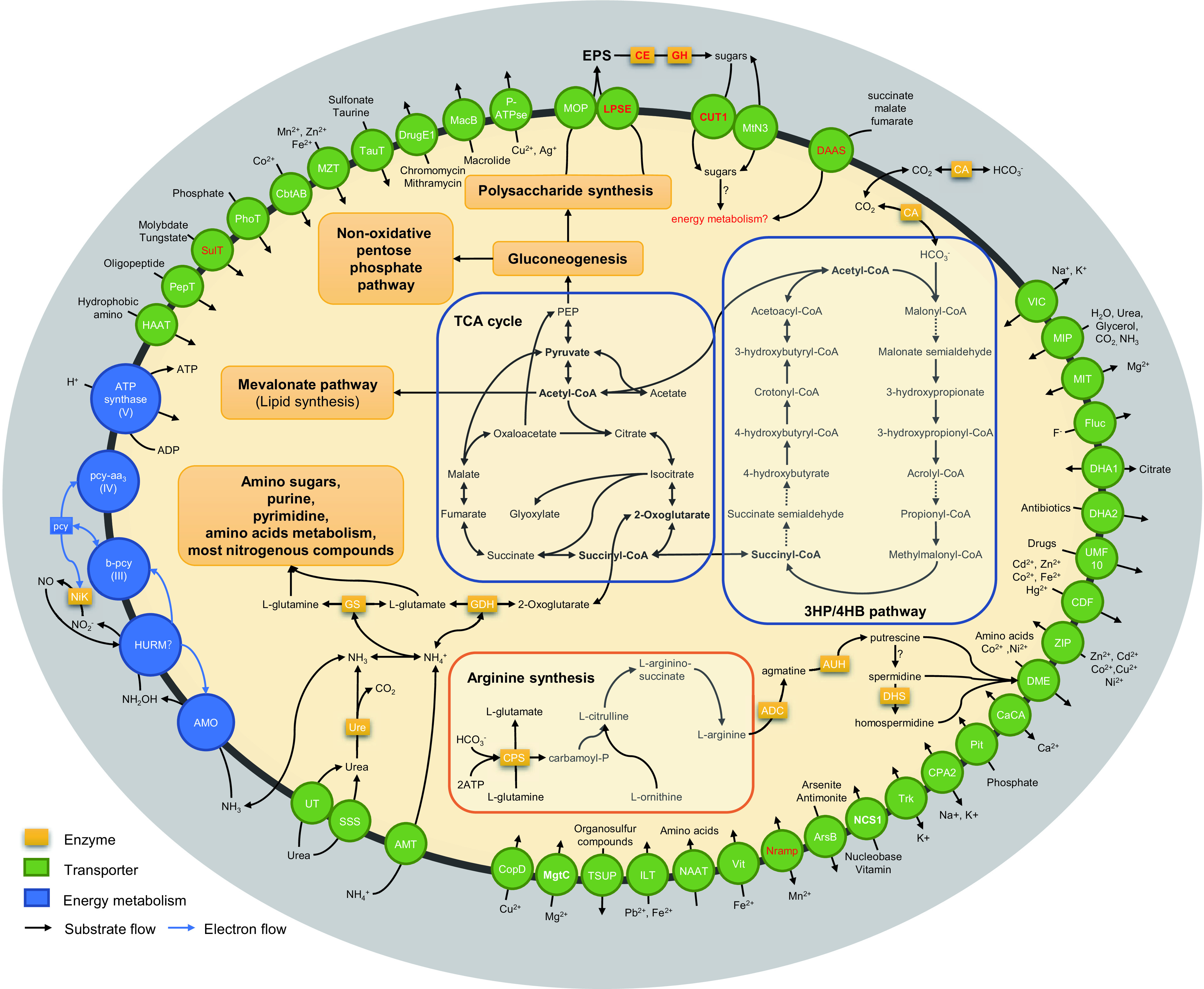
Schematic reconstruction of the predicted metabolic modules and other genome features of “*Ca.* Nitrosocosmicus agrestis.” Dashed lines indicate reactions for which the enzymes have not been identified. CE, carbohydrate esterase; GH, glycoside hydrolase; CA, carbonic anhydrase; NirK, nitrite reductase; Ure, urease; GDH, glutamate dehydrogenase; GS, glutamine synthetase; ADC, arginine decarboxylase; AUH, agmatinase; DHS, deoxyhypusine synthase. The unique enzymes or transporters of *Nitrosocosmicus* clade are in red. Candidate enzymes, gene accession numbers, and transporter classes are listed in the supplemental tables.

A genome of “*Ca.* Nitrosocosmicus agrestis” was recovered from a metagenomic assembly and contains 43 contigs with a total length of 3.22 Mbp (Fig. S4). The completeness, contamination, and strain heterogeneity of the genome were 96.1%, 2.91% and 0%, respectively. The genome has an average G+C content of 33.42%, includes 3,513 protein-coding sequences (CDS), and contains 45 tRNA genes, one 5S rRNA gene, and two 16S/23S rRNA operons (Table 2). The genes involved in central carbon metabolism, including autotrophic CO_2_ fixation (3HP/4HB pathway), tricarboxylic acid cycle, gluconeogenesis, and nonoxidative pentose phosphate pathways, are presented (Table S1; Fig. 4). Genes encoding pathways of ammonia oxidation (single copies of *amoA*, *amoB*, and *amoX* and three copies of *amoC*) and urea hydrolysis (urease subunits, urea accessory proteins, and urea transporter), which participate in energy metabolism, are also found in the genome (Table S1; Fig. 4). Urease utilization genes were always found in the terrestrial genera “*Ca.* Nitrosocosmicus” and *Nitrososphaera* and sometimes in the marine genus *Nitrosopumilus* (Table 2).

**TABLE 2:** Genome features of “*Ca.* Nitrosocosmicus agrestis” and other AOA

AOA strain	Genome size (Mb)	GC (%)	No. of protein coding genes	Coding region (%)	No. of:		Motility	Urease	No. of genes for:					
					rRNA operons	tRNA genes			β-CA	γ-CA-Cam	γ-CA-CamH	Amt-1	Amt-2	DME^*a*^
“*Ca.* Nitrosocosmicus agrestis” SS	3.22	33.41	3,524	70.74	2	45	−	+	1	1	1	−	1	4 (3)
“*Ca.* Nitrosocosmicus exaquare” G61	2.99	33.94	3,196	77.32	2	36	−	+	−	2	1	−	1	4 (2)
“*Ca.* Nitrosocosmicus oleophilus” MY3	3.43	34.14	3,722	73.96	3	38	−	+	1	3	1	−	1	2 (2)
“*Ca.* Nitrosocosmicus franklandus” C13	2.84	34.07	3,025	72.94	2	39	−	+	−	1	1	−	1	3 (3)
“*Ca.* Nitrosocosmicus arcticus” Kfb	2.64	34.00	2,970	75.83	3	33	−	+	1	1^*b*^	1	−	1	3 (1)
*N. viennensis* EN76	2.53	52.72	3,123	87.32	1	39	+	+	−	1	−	2	1	1
“*Ca.* Nitrososphaera gargensis” Ga9.2	2.83	48.35	3,562	81.53	1	37	+	+	−	−	−	2	1	1
“*Ca.* Nitrososphaera evergladensis” SR1	2.95	50.14	3,499	83.60	1	39	+	+	−	1	−	2	1	1
“*Ca.* Nitrosotalea devanaterra” Nd1	1.81	37.07	2,067	90.99	1	41	+	−	−	1	−	1	2	1
“*Ca.* Nitrosotenuis aquarius” AQ6f	1.70	42.18	2,008	92.90	1	42	+	+	−	−	−	1	1	1
“*Ca.* Cenarchaeum symbiosum” A	2.05	57.37	2,017	91.67	1	45	−	+	−	−	−	2	−	−
“*Ca.* Nitrosopelagicus brevis” CN25	1.23	33.16	1,469	94.48	1	42	−	−	−	−	−	1	1	−
“*Ca.* Nitrosoarchaeum limnia” SFB1	1.74	32.46	2,038	84.83	1	45	+	−	−	−	−	1	1	2
“*Ca.* Nitrosomarinus catalina” SPOT01	1.36	31.44	1,677	92.48	1	43	−	+	−	−	−	1	1	−
*N. maritimus* SCM1	1.65	34.17	1,797	90.82	1	40	−	−	−	−	−	1	1	2
Nitrosopumilus adriaticus NF5	1.80	33.41	2,206	90.61	1	40	+	−	−	−	−	1	1	1
Nitrosopumilus piranensis D3C	1.71	33.82	2,140	90.37	1	41	−	+	−	−	−	1	1	3
“*Ca.* Nitrosocaldus islandicus” 3F	1.62	41.54	1,746	87.67	1	39	+	+	−	−	−	2	1	−

a The number in parentheses indicates the number of copies of the putative polyamine exporter gene.

b The complete gene sequence cannot be obtained by gene prediction.

10.1128/mSystems.01003-20.4 FIG S4 Classification and coverage of metagenome contigs (A) and composition of bins from the “*Ca.* Nitrosocosmicus agrestis” cultures (B); bin 2 is the genome of “*Ca.* Nitrosocosmicus agrestis,” bin 3 is the genome of *Ensifer* sp., and bin 4 consists of contigs of “*Ca.* Nitrosocosmicus” but did not pass the quality check of CheckM. Download FIG S4, TIF file, 0.3 MB. Copyright © 2021 Liu et al. 2021 Liu et al. https://creativecommons.org/licenses/by/4.0/ This content is distributed under the terms of the Creative Commons Attribution 4.0 International license.

10.1128/mSystems.01003-20.8 TABLE S1 Functional genes for carbon metabolism, energy metabolism, transporters, carbohydrate-active enzyme, and nitrogen metabolism in the “*Ca.* Nitrosocosmicus agrestis” genome and genes for polyamine metabolism and transport in the coexisting heterotroph *Ensifer* sp. strain SSB1. Download Table S1, XLSX file, 0.04 MB. Copyright © 2021 Liu et al. 2021 Liu et al. https://creativecommons.org/licenses/by/4.0/ This content is distributed under the terms of the Creative Commons Attribution 4.0 International license.

During carbon metabolism in AOA, the first step is the fixation of inorganic carbon into cells via the 3HP/4HB pathway, with the help of carbonic anhydrase (CA), which is responsible for the reversible hydration of CO_2_ to HCO_3_^−^ (35, 36). Three genes encoding CA were identified in the genome of “*Ca.* Nitrosocosmicus agrestis” (Table 2; Table S1); they were assigned to the β class (d clade) and γ class (Cam and CamH) according to the phylogenetic analysis (Fig. S5). Until now, the CA genes had been identified only in the genomes of terrestrial AOA strains from the genera “*Ca.* Nitrosocosmicus,” *Nitrososphaera*, and “*Ca.* Nitrosotalea” (Table 2). In contrast to the periplasmic location of Cam, CamH and β-CA are located in the cytosol in the absence of signal peptide. Meanwhile, a full set of genes encoding the key enzymes and transporters that participate in polysaccharide metabolism were also found in the genome, including members of the glycosyltransferase (GT) family, members of the glycoside hydrolase (GH) family, members of the carbohydrate esterase (CE) family, multidrug/oligosaccharidyl-lipid/polysaccharide (MOP) flippase, members of the lipopolysaccharide exporter (LPSE) family, etc. (Table S1) (37). These proteins are important for cell surface modification and exopolysaccharide (EPS) production of biofilm-forming bacteria and archaea. An extensive set of genes encoding these proteins was identified among the AOA groups from the *Nitrososphaerales*, such as Nitrososphaera viennensis, “*Ca.* Nitrososphaera gargensis,” and “*Candidatus* Nitrososphaera evergladensis” (37). Interestingly, the genes encoding LPSE are unique among the genomes of genus “*Ca.* Nitrosocosmicus,” suggesting the potential of polysaccharide secretion for this AOA clade.

10.1128/mSystems.01003-20.5 FIG S5 Evolution analysis of β (A) and γ (B)-carbonic anhydrase based on amino acid sequence. The software was IQ-TREE, the method used was maximum likelihood, the model was LG+I+G4, and the ultrafast bootstrap value was 1,000. Download FIG S5, TIF file, 1.7 MB. Copyright © 2021 Liu et al. 2021 Liu et al. https://creativecommons.org/licenses/by/4.0/ This content is distributed under the terms of the Creative Commons Attribution 4.0 International license.

During nitrogen metabolism, the ammonia nitrogen that diffuses through cytoplasmic membrane (in the form of NH_3_) or is transferred from the extracellular environment by ammonium transporters (Amt) (in the form of NH_4_^+^) is integrated into glutamine/glutamate syntheses. A single copy of the gene encoding Amt was identified in the genome of “*Ca.* Nitrosocosmicus agrestis” (Table 2; Fig. S6A; Table S1). Based on the phylogenetic analysis, it was assigned to Amt-2, a cluster of Amt showing low affinity to NH_4_^+^ (Fig. S6A); the ammonium-binding site of Amt-2 is as conserved as that of Amt-1 (Fig. S6B), suggesting their specific binding to ammonium. The known strains of “*Ca.* Nitrosocosmicus” were found to have only one copy of the Amt-2 gene in their genomes; however, almost all of the other AOA strains have genes for two types of Amt in their genomes, the high-affinity Amt-1 and the low-affinity Amt-2 (Table 2; Fig. S6B). Besides the genes encoding glutamine synthetase (GS) and glutamate dehydrogenase (GDH), the genes encoding type II carbamoyl phosphate synthetase (CPS) were also found in the genome (Table S1), suggesting the organisms’ ability to hydrolyze glutamine to glutamate and produce carbamoyl phosphate as a by-product (Fig. 4) (38). Along with CPS, the key enzymes, involved in arginine synthesis, including ornithine transcarbamylase (OTC), argininosuccinate synthase (ASS) and argininosuccinate lyase (ASL), were also found in “*Ca.* Nitrosocosmicus agrestis” (Table S1). When arginine was used as the substrate, it could be further catalyzed to polyamines via the activities of arginine decarboxylase (ADC), agmatinase (AUH), spermidine synthase (SDS), and deoxyhypusine synthase (DHS). Except for the SDS gene, these genes were identified in the genome of “*Ca.* Nitrosocosmicus agrestis,” which suggests that arginine could be converted into putrescine and some other polyamines after a series of enzymatic catalysis steps (Fig. 4).

10.1128/mSystems.01003-20.6 FIG S6 Evolution analysis of Amt amino acid sequences in the different AOA genomes (A). The software was IQ-TREE, the method used was maximum likelihood, the model was LG+F+R3, and the ultrafast bootstrap value was 1,000. Sequence alignment of the AOA Amt protein to the *N. europaea* Rh protein based on the program MUSCLE (B). The residues that constitute the proposed ammonium-binding site are marked with a red asterisk. Download FIG S6, TIF file, 2.8 MB. Copyright © 2021 Liu et al. 2021 Liu et al. https://creativecommons.org/licenses/by/4.0/ This content is distributed under the terms of the Creative Commons Attribution 4.0 International license.

The genes encoding the pathways of arginine synthesis as well as polyamine synthesis are found in most of the AOA genomes, suggesting their importance for amino acid synthesis or stress responses among AOA species. Although significant functions of polyamine have been reported for bacteria and archaea (39), excessive accumulation of polyamines and the related metabolites in intracellular spaces likely causes toxic effects (40). Therefore, an effective means of secreting the synthesized polyamine from the cells is required, or the polyamine synthesis would be repressed. Unfortunately, no gene has been annotated as the transporter protein or efflux pump used for the excretion of polyamines in AOA. In some bacteria and yeasts, members of the drug/metabolite exporter (DME) family have been reported to be able to mediate the excretion of amino acids and polyamines (41, 42). Based on sequence analysis, four copies of the gene encoding DME were identified in the genome of “*Ca.* Nitrosocosmicus agrestis” (Fig. S7A). In fact, the genes encoding DME were found to be prevalent among the AOA genomes (Table 2). According to phylogenetic analysis, these DMEs were clustered into three separate groups, with the sequences from “*Ca.* Nitrosocosmicus” in two groups and the sequences from other genera, including “*Ca.* Nitrosopumilus,” “*Ca.* Nitrosotalea,” *Nitrososphaera*, “*Ca.* Nitrosotenuis,” “*Ca.* Nitrosoarchaeum,” and “*Ca.* Nitrosocosmicus,” in one group (Fig. S7A). Interestingly, one DME group belonging to the genus “*Ca.* Nitrosocosmicus” are annotated as the probable exporters of amino acids or other metabolites, while the DMEs from other AOA genera are annotated as the Co^2+^/Ni^2+^ efflux porter or uncharacterized; moreover, the phylogenetic analysis indicated that the DMEs of “*Ca.* Nitrosocosmicus” are closely related to the DMEs of *Escherichia* and *Saccharomyces* (Fig. S7B), which have been identified to act as the efflux pump of polyamines (43, 44).

10.1128/mSystems.01003-20.7 FIG S7 Evolution analysis of DME transporter in the different AOA genomes (A) and evolution analysis of polyamine exporters and AOA DME transporters (B). The functionally identified polyamine exporters in bacteria and yeasts are in blue; the speculated polyamine exporters in “*Ca.* Nitrosocosmicus” are in red; the codes in parentheses are accession numbers in UniProt or NCBI. The software was IQ-TREE, the method was maximum likelihood, the model was LG+F+R4, and the ultrafast bootstrap value was 1,000. Percent identity between protein sequences of DME1, DME2, DME3, and DME4 in “*Ca.* Nitrosocosmicus agrestis” (C). The method was BLASTp, and the matrix was BLOSUM45. Download FIG S7, TIF file, 1.1 MB. Copyright © 2021 Liu et al. 2021 Liu et al. https://creativecommons.org/licenses/by/4.0/ This content is distributed under the terms of the Creative Commons Attribution 4.0 International license.

### Gene expression and protein and polyamine production of “*Ca.* Nitrosocosmicus agrestis” at high ammonia concentrations.

**FIG 5:**
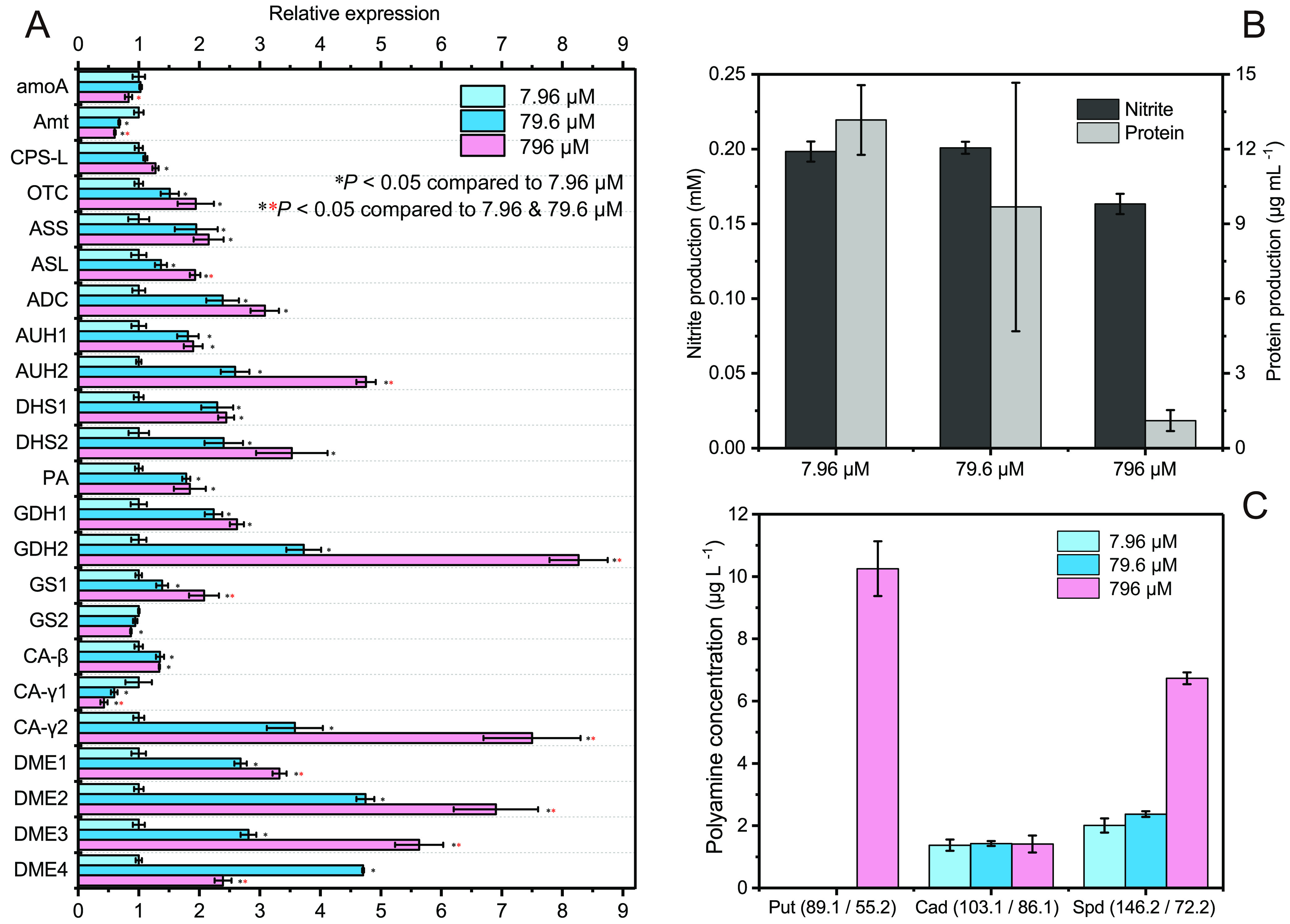
Metabolic characteristics of “*Ca.* Nitrosocosmicus agrestis” at different un-ionized-ammonia concentrations. (A) Relative gene expression levels in response to high ammonia. RNA was extracted from triplicate pooled cultures. Error bars indicate standard errors of the means for technical triplicates of qPCR. (B) Nitrite production and protein synthesis by “*Ca.* Nitrosocosmicus agrestis” in 2 days with 7.96 to 796 μM ammonia. (C) Effect of ammonia on the accumulation of polyamine by “*Ca.* Nitrosocosmicus agrestis.” Put, putrescine; Cad, cadaverine; Spd, spermidine. Error bars indicate standard errors of the means for biological triplicates. RNA extraction and protein and polyamine quantification were carried out after the addition of 7.96 to 796 μM un-ionized ammonia for 2 days.

In comparison with the gene expression levels at a low ammonia concentration (7.96 μM NH_3_), the expression patterns of genes involved in ammonia oxidation, arginine synthesis, polyamine synthesis, glutamine/glutamate syntheses, carbonic anhydrase, and polyamines transport when “*Ca.* Nitrosocosmicus agrestis” responded to high ammonia had significant changes. For example, *amoA* and the Amt gene were significantly downregulated at 796 μM NH_3_; however, all of the genes participating in arginine synthesis, polyamine synthesis, and polyamine excretion had significant upregulation, with values ranging from 1.28 to 5.63 times (Fig. 5A). The genes encoding GDH, GS and intracellular CA (β-CA and CamH of γ-CA) were also significantly upregulated; for the genes encoding GDH2 and CA-γ2, expression levels were about 8.3 and 7.5 times higher, respectively (Fig. 5A).

During chemoautotrophic growth of “*Ca.* Nitrosocosmicus agrestis” using ammonia as the sole energy source, the energy generated from ammonia oxidation is mainly used for inorganic carbon fixation, biomass production, and life maintenance. During growth of “*Ca.* Nitrosocosmicus agrestis” with 7.96, 79.6, and 796 μM ammonia, the protein production rates were determined to be 13.16, 9.68, and 1.11 μg ml^−1^, respectively (Fig. 5B). The average ammonia oxidation rate of “*Ca.* Nitrosocosmicus agrestis” exposed to high ammonia (796 μM NH_3_) decreased by 17% in contrast to that seen with low levels of ammonia (7.96 μM NH_3_) (Fig. 5B); however, the protein synthesis efficiency decreased by 89.8% (Fig. 5B). It is suggested that a large amount of energy was used for syntheses of other compounds, such as polyamines, in order to tolerant high ammonia levels.

The concentrations of putrescine, cadaverine, spermidine, and spermine in the supernatant of the “*Ca.* Nitrosocosmicus agrestis” cultures grown with different concentrations of ammonia were determined by using high-performance liquid chromatography-tandem mass spectrometry (HPLC-MS/MS). Polyamines including putrescine, cadaverine, and spermidine were identified, with concentrations ranging from 1.37 to 10.25 μg liter^−1^ (Fig. 5C). Relative to the rates of production in the presence of a low ammonia concentration (7.96 μM NH_3_), cadaverine production was not significantly different but spermidine production was about three times higher; notably, putrescine was detected only with a high level of ammonia (796 μM NH_3_) and was identified as the predominant polyamine in the culture (Fig. 5C). Putrescine is usually regarded as the precursor for the syntheses of other kinds of polyamines (45). The accumulation of putrescine in the presence of high ammonia concentrations suggested that polyamine synthesis could be a strategy of the “*Ca.* Nitrosocosmicus agrestis” response to ammonia stress.

## DISCUSSION

The terrestrial environments of agricultural soils are usually more variable and complex than marine environments. This requires the terrestrial AOA to evolve different strategies to survive in these environments. Generally, the genomes of terrestrial AOA are much larger than those of the marine AOA (Table 2); they have evolved and obtained more genes, probably in order to encode more proteins in response to different kinds of environmental stresses. For example, genes encoding the pathways of polysaccharide metabolism, such as the synthesis enzymes (GTs, GHs, and CEs) and transporter proteins (MOP and LPSE), are prevalent among the genomes of terrestrial AOA; they probably synthesize polysaccharide to modify the cell surface and then form biofilms, which provides protection and promotes survival in rapidly changing environmental conditions, such as fixation on solid matrices, cooperation with bacteria, and tolerance to high levels of ammonia.

The newly nominated genus “*Ca.* Nitrosocosmicus” is an important part of the AOA community in agricultural soils (8, 46, 47); though low in numbers, they contribution more to ammonia oxidation than bacterial oxidizers (47, 48). The known species of “*Ca.* Nitrosocosmicus” are able to grow at NH_3_ concentrations that inhibit other cultivated AOA (Table 1), reflecting their identity as ammonia-tolerant AOA groups. Due to the presence of this kind of AOA group, AOA-mediated ammonia oxidation and greenhouse gas N_2_O production in soils were not completely suppressed by high ammonia (18, 49). The finding of “*Ca.* Nitrosocosmicus” indicates that ammonia toxicity does not clearly differentiate AOA from AOB. It is well known that AOB species have high tolerance to ammonia; however, the related mechanism has rarely been reported. The rhesus (Rh) proteins, belonging to the Amt/MEP/Rh (ammonium transporter/methylammonium-ammonium permease/rhesus protein) family and acting as bidirectional transporters of NH_4_^+^/NH_3_, have been reported in *Nitrosomonas* (50, 51). They could mediate ammonia efflux, which then reduces the NH_3_ toxicity, making it possible for them to detoxify ammonia for fish (52). Unfortunately, the gene encoding Rh protein or related protein is absent in the AOA genome; the AOA seem to prefer to use Amt as the transporter of NH_4_^+^. Some different mechanisms for the ammonia tolerance are probably used by the AOA species of the genus “*Ca.* Nitrosocosmicus.”

**FIG 6:**
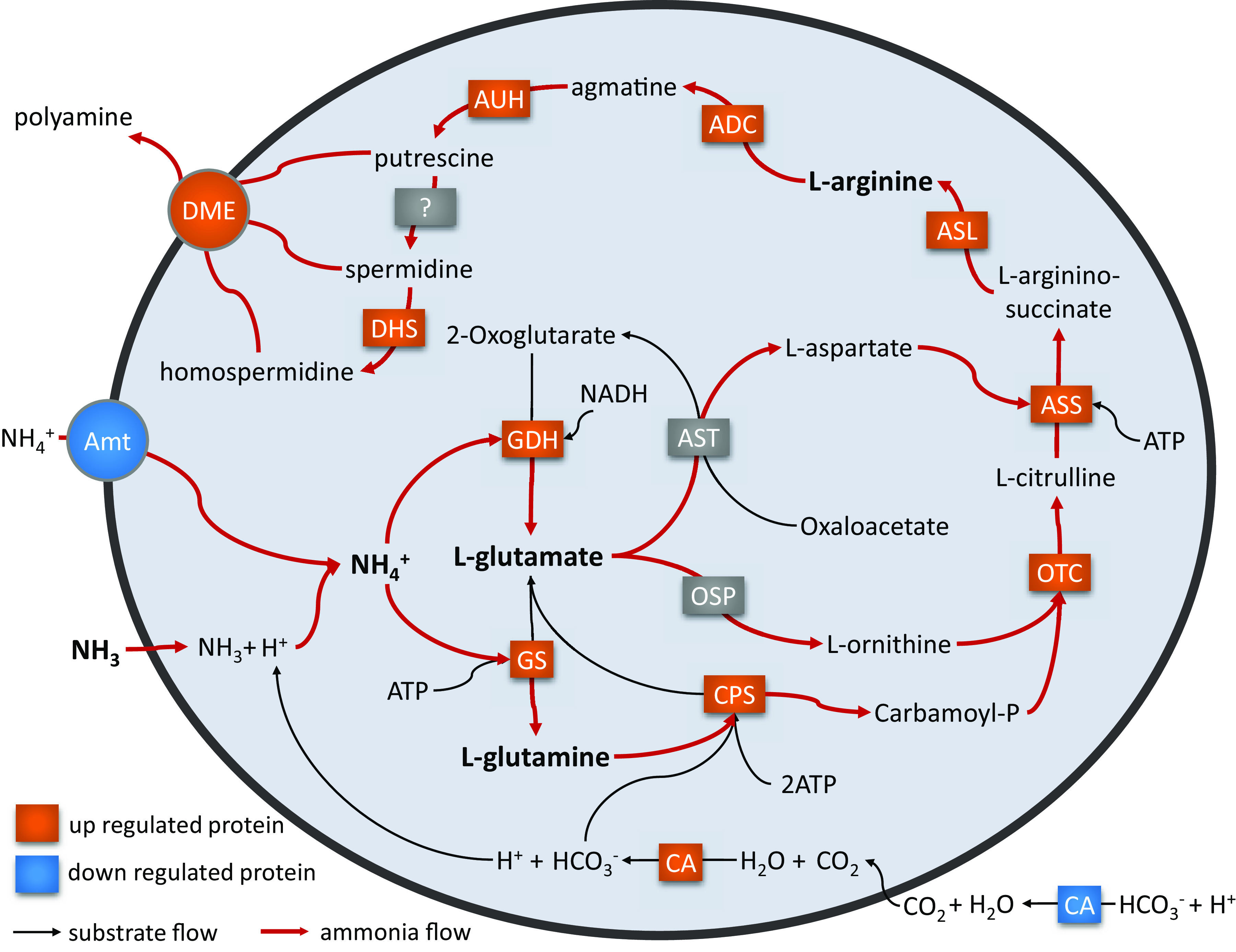
Schematic mechanism of ammonia tolerance of “*Ca.* Nitrosocosmicus agrestis.” The red arrows represent the flow of ammonia nitrogen. *amoA*, ammonia monooxygenase subunit A; Amt, ammonium transporter; CPS, carbamoylphosphate synthase large subunit; OTC, ornithine carbamoyltransferase; ASS, argininosuccinate synthase; ASL, argininosuccinate lyase; ADC, arginine decarboxylase; AUH, agmatinase; DHS, deoxyhypusine synthase; PA, putrescine aminotransferase; GDH, glutamate dehydrogenase; GS, glutamine synthetase; DME, drug/metabolite exporter family; CA, carbonic anhydrase.

In this study, an AOA strain “*Ca.* Nitrosocosmicus agrestis” obtained from agricultural soil was determined to have high tolerance to ammonia (Fig. 3). According to studies on comparative genomic analysis, gene expression quantification, and protein and extracellular metabolite determination, the potential mechanisms of “*Ca.* Nitrosocosmicus agrestis” and possibly the genus “*Ca.* Nitrosocosmicus” that allow tolerance of high ammonia were proposed, as follows (Fig. 6).

The first notable finding in this regard is the presence of only the Amt-2 transporter in genomes (Table 2; Fig. S6); it shows low affinity to NH_4_^+^ and would be significantly downregulated in response to high ammonia (Fig. 5A), suggesting that entrance of ammonia into the cytoplasm occurs mainly by spontaneous diffusion. In general, the ammonia concentrations in terrestrial habitats are usually higher than those in the ocean, which carries the evolutionary implication that the terrestrial AOA do not need the high-affinity ammonium transporter Amt-1, because the amount of NH_3_ diffused across the inner membrane is sufficient for their metabolism. As a result of this evolutionary step, the ammonia tolerance of terrestrial AOA would be higher than that of the marine AOA. With respect to the mechanism, the NH_3_ that diffuse through inner membrane is combined with H^+^ (derives from the chemical equilibrium: H_2_CO_3_ ⇌ HCO_3_^−^ + H^+^) to form NH_4_^+^. However, if it presents Amt-1, NH4^+^ transport into the cytoplasm via Amt-1 would be the main way for ammonia to enter cells. At this stage, the assimilation of NH4^+^ and bicarbonate will produce a large amount of H^+^, which in turn will decrease the cytoplasmic pH and significantly suppress cell growth.

Second, cytoplasmic CAs are unique to the genus “*Ca.* Nitrosocosmicus” (Table 2) and are significantly upregulated in response to high ammonia (Fig. 5A); they are capable of supplying plenty of HCO_3_^−^ for the 3HP/4HB pathway and arginine synthesis, as well as supplying H^+^ for the conversion of NH_3_ to NH_4_^+^.

The third mechanism is the presence of an incomplete ornithine cycle pathway in the genome of “*Ca.* Nitrosocosmicus agrestis.” Due to the lack of arginase, the arginine that is synthesized via the pathway mediated by the enzymes CPS, OTC, ASS, and ASL could not be catalyzed to ornithine and urea and then be used as the precursor for polyamine synthesis. The genes that encode the enzymes involved in glutamine (as the substrate for CPS-II) and arginine syntheses were significantly upregulated under high-ammonia conditions, which suggested that the NH_3_ that diffuses through the cell membrane was probably used for arginine synthesis and further polyamine synthesis.

Fourth, “*Ca.* Nitrosocosmicus agrestis” probably uses DME as an exporter of polyamine. The DME genes of “*Ca.* Nitrosocosmicus agrestis” that are closely related to the bacterial putative DME were determined to be upregulated 2.5- to 7.5-fold when growing with high levels of ammonia (Fig. 5A), and the production of extracellular polyamines were also much higher (Fig. 5C), suggesting that polyamines were not only synthesized in cells but also excreted from the cells. As a result, the maintenance of cytoplasmic pH and production of polyamine are probably responsible for the ammonia tolerance of “*Ca.* Nitrosocosmicus agrestis” and for other species of “*Ca.* Nitrosocosmicus.”

Polyamines are a class of small cation that contain multiple amino groups. They play important roles in nucleic acid stabilization, protein translation, stress responses, and cell division (39). However, high intracellular accumulation of polyamines is very harmful to the cells. For example, excess polyamines in the cytoplasm are likely to interfere with cellular metabolism, including protein synthesis, and the oxidative decomposition of polyamines generates reactive oxygen species (ROS) that are highly damaging to cells (40). Because the positively charged polyamines are unable to diffuse through cell membranes by themselves, certain types of transporter are needed to excrete them from the cells. The DME family, belonging to the DMT superfamily, has been reported to be able to export various compounds such as amino acids and polyamines to maintain intracellular homeostasis (53, 54). Some marine AOA can exude a suite of organic nitrogen-containing compounds, including amino acids, thymidine, and B vitamins, out of cells to fuel prokaryotic heterotrophs or release labile organic matter to recruit H_2_O_2_-detoxifying heterotrophs to self-rescue (55, 56). Unfortunately, no related transporter of amino acid was identified in the genus “*Ca.* Nitrosocosmicus.” Though the genes encoding DME protein are ubiquitous in the genomes of AOA, the function of DME in the excretion of amino acids among these marine AOA needs further research. In this study, the amino acid in the supernatant of “*Ca.* Nitrosocosmicus agrestis” growing with low or high concentrations of ammonia is almost undetectable. Other than the excreted amino acid being used by its bacterial consortium in cultures, there is no evidence at present to prove that “*Ca.* Nitrosocosmicus agrestis” could exude amino acid.

It is also suggested that the excretion of amino acids (especial the glutamate group of amino acids) from cells would not be a mechanism of ammonia tolerance. However, polyamines, including putrescine, cadaverine, and spermidine, were identified in the supernatant of “*Ca.* Nitrosocosmicus agrestis,” and their concentrations increased along with the upregulation of DME genes under high-ammonia conditions (Fig. 5C), suggesting the possibility of DME acting as the transporter of polyamines. According to the phylogenetic analysis, a certain DME of “*Ca.* Nitrosocosmicus” is distinct from that of other AOA and closely related to the polyamine exporter of bacteria and yeasts (Fig. S7). It is proposed that the DME could potentially act as an exporter of polyamines in “*Ca.* Nitrosocosmicus agrestis.”

Inexplicably, the polyamines detected in the cultures of “*Ca.* Nitrosocosmicus agrestis” grown with either a high or low concentration of ammonia were far from corresponding to the amount of ammonia oxidized. Several explanations for this observation are possible. First, the role of polyamines is probably as an active molecule, like their functions in bacteria and archaea (39); a low concentration of polyamine was enough to help AOA cells to tolerate high ammonia, which implies that there is therefore no need to synthesize large amounts of polyamines to decrease the concentration of ammonia and reduce ammonia toxicity. Second, the culture of “*Ca.* Nitrosocosmicus agrestis” was not pure, as it comprised about 5% (much more in the presence of high ammonia) bacteria belonging to the *Rhizobiales* (Fig. S3); therefore, the polyamines synthesized and secreted by AOA cells were immediately used to fuel the growth of heterotrophic bacteria. Based on the metagenome data of “*Ca.* Nitrosocosmicus agrestis” culture, one genome assigned to *Ensifer* sp. strain SSB1 was recovered, which represented the predominant bacterial partner in the culture (Fig. S4B; Table S1). The genome of *Ensifer* sp. strain SSB1 contains 14 copies of *potABCD* and one of *ydcSTUV* (Table S1), which have been reported to encode the polyamine transporters used for polyamine uptake (57). The gene copy number of *potABCD* is much higher than in the related strains Ensifer adhaerens OV14 (4 copies) and Ensifer adhaerens Casida A (6 copies) (58, 59). In addition, the genome of *Ensifer* sp. strain SSB1 also contains several different coding genes responsible for polyamine degradation (Table S1) (60, 61). The presence of multiple copies of polyamine transporter and metabolism genes indicates that *Ensifer* sp. strain SSB1, as well as some other coexisting heterotrophic bacteria, could probably use the polyamines as an organic carbon source. Third, at high concentrations of ammonia, ammonia nitrogen entered the cytoplasm mainly by spontaneous diffusion and not by membrane-crossing transport via Amt, which makes the concentration of intracellular ammonia much lower than that of extracellular ammonia. Therefore, the synthesis of a small amount of polyamines from intracellular ammonia is enough to reduce the ammonia toxicity.

The polyamines were almost certainly present in the cells of the last universal common ancestor (LUCA) of all life and widely distributed among the hyperthermophilic, acidophilic, and thermoacidophilic archaea, suggesting their potential role in the response to adverse environmental conditions (39). The polyamine structures in different microbial species are diverse, as are their lifestyles, which makes many of them are still unknown. Moreover, microbial interactions are widespread in nature. The relationship between the genus “*Ca.* Nitrosocosmicus” and heterotrophs has barely been reported, and so some unknown or complex interactions between them may occur, with the result that many terrestrial AOA species are uncultivated or even unclassified (62). The polyamine-mediated interaction between AOA and bacteria would be most likely to expand the ecological role of AOA in nature. This case study was performed on only one strain from the genus “*Ca.* Nitrosocosmicus.” In order understand why “*Ca.* Nitrosocosmicus agrestis” or the genus “*Ca.* Nitrosocosmicus” is able to tolerate high ammonia levels, additional work on more AOA strains is needed to elucidate whether the polyamine is responsible for ammonia tolerance and what happens between “*Ca.* Nitrosocosmicus” and its bacterial consortia.

## MATERIALS AND METHODS

### AOA strain and culture maintenance.

“*Ca.* Nitrosocosmicus agrestis” strain SS was obtained from agricultural soil by using a two-step strategy and maintained in a mineral salts medium (MSM) (5). The culture was incubated at 30°C in the dark without shaking; an antibiotic mixture containing ciprofloxacin (50 mg/liter), azithromycin (50 mg/liter), and natamycin (10 mg/liter) was added to the culture to maintain a high abundance of archaeal cells. Ten percent (wt/vol) quartz (1 mm in diameter) was supplied for the attachment of archaeal cells during the seed cultivation.

### Physiological characterization.

AOA cells attached to quartz were washed out by vortex-shaking in fresh liquid MSM; the suspension with a rate of 10% (vol/vol) was inoculated into the fresh MSM without supplying quartz and subjected to the determination of physiological characteristics. To avoid pH deviation due to hydrolysis or dissociation of bicarbonate, the MSM used for pH characterization was not supplied with NaHCO_3_ (the diffused CO_2_ is sufficient for SS growth) but was buffered with 10 mM MES [2-(*N*-morpholino)ethanesulfonic acid], HEPES, or Tris to maintain the pH in the ranges of 6 to 7, 7 to 8, and 8 to 9, respectively. Concentrations of nitrite and ammonium were determined by the standard Griess-Ilosvay method and the indophenol blue method, respectively (63). The concentration of un-ionized ammonia was calculated using the formula of Emerson et al. (64). The mean ammonia oxidation rate was calculated basing on the determination of nitrite concentration. The abundance of AOA and bacteria in the cultures was analyzed using absolute quantitative PCR based on 16S rRNA gene primers (SS16S-1F/SS16S-1R for “*Ca.* Nitrosocosmicus agrestis” and 1369F/1492R for bacteria), following the method described in our previous study (5).

### Microscopy assays.

Fluorescence *in situ* hybridization (FISH) was observed on a scanning confocal microscope (LSM 710; Carl Zeiss, Germany); transmission electron microscopy (TEM) was performed on an H-7650 transmission electron microscope (Hitachi, Tokyo, Japan) at an accelerating voltage of 80 kV; scanning electron microscopy (SEM) was performed using a Merlin field emission scanning electron microscope (Carl Zeiss, Jena, Germany).

### Genome sequencing, assembly, and annotation.

The genomic DNA from 3,000 ml batch cultures was extracted according to a previously described protocol (5). Purified DNA was fragmented to ∼400 bp with the aid of an M220 focused ultrasonicator (Covaris, Woburn, MA, USA) and subsequently used for library preparation using a TruSeq DNA sample prep kit (Illumina, San Diego, CA, USA). Metagenomic sequencing was performed on a HiSeq X Ten sequencing system (Illumina, San Diego, CA, USA).

The raw data were length and quality filtered using Trimmomatic v0.38 (65) and *de novo* assembled using metaSPAdes v3.13.0 (66) with 127-mers. The GapCloser module of SOAPdenovo2 v1.12 (67) was used with default parameters to fill a proportion of gaps of the assembled scaffolds. Genome binning of the metagenomic assemblies was conducted using MaxBin2 v2.26 (68) with the universal marker gene sets (69). Scaffolds in the assembly with lengths of <1,000 bp were removed. The completeness, contamination, and strain heterogeneity of genome were evaluated using CheckM v1.013 (70). Gene prediction and annotation were done using JGI-IMG/MER (71) and MicroScope platform (72). KEGG pathways were generated using KASS (73). Putative transport proteins and carbohydrate-active enzymes were identified using TCDB (74) and dbCAN2 (75), respectively. The average nucleotide identity (ANI) and average amino acid identity (AAI) of five *Nitrosocosmicus* genomes were calculated using JSpeciesWS (76) and compareM (https://github.com/dparks1134/CompareM), respectively.

A phylogenomic tree was obtained using the automatically generated alignment of 43 concatenated universal marker protein sequences, which were identified by CheckM v1.013 (70). The best-fit model of evolution was selected with ModelFinder (77), and phylogenomic trees were inferred by maximum likelihood with IQ-TREE v1.6.1 (78).

### Relative transcriptional expression quantitative analysis.

During the total RNA extraction, AOA cells in cultures were collected by filtering through a 0.22-μm cellulose filter; the filter was cut into pieces and ground in a tube containing 0.5 g quartz sand. RNA extraction was performed using RNAiso Plus (TaKaRa, Dalian, China); the synthesis of first-strand cDNA was performed using the PrimeScript RT reagent kit with gDNA Eraser (TaKaRa, Dalian, China). Quantitative PCR were performed on an ABI 7500 Fast real-time PCR system (Applied Biosystems, Carlsbad, CA, USA) using TransStart Tip Green qPCR SuperMix (Transgen, Beijing, China). The reaction conditions were as follows: 1 min at 94°C and 40 cycles of 10 s at 94°C and 34 s at 60°C. The 2^−ΔΔ^*^CT^* method was used to estimate fold change in gene expression, normalized to the endogenous control 16S rRNA. Primer pairs (Table S2) were designed on the function genes from the “*Ca.* Nitrosocosmicus agrestis” genome. All experiments were prepared in triplicate.

10.1128/mSystems.01003-20.9 TABLE S2 Primers used for quantitative PCR. Download Table S2, XLSX file, 0.01 MB. Copyright © 2021 Liu et al. 2021 Liu et al. https://creativecommons.org/licenses/by/4.0/ This content is distributed under the terms of the Creative Commons Attribution 4.0 International license.

### Protein determination.

To measure the total protein in AOA cells, about 20 ml of culture was collected and filtered through a 0.22-μm cellulose filter. The filter with cells was cut into pieces and ground in a 2-ml tube containing 0.5 g quartz sand, and 500 μl of lysis buffer (TE buffer containing 1% Triton X-100) was added to the tube and mixed for 5 min using the Vortex Adapter (13000-V1-24; Qiagen, Germany). After centrifugation at 5,000 × *g* for 5 min, the pellet was collected and used for protein measurement by using a bicinchoninic acid (BCA) protein assay kit (Sangon Biotech, Shanghai, China).

### Quantification of polyamines and amino acids in supernatant.

An 800-μl portion of the supernatant was collected and filtered through a 0.22-μm cellulose filter. The filtrate was mixed with 200 μl of sulfosalicylic acid (10%) and incubated at 4°C for 1 h, following centrifugation at 14,000 × *g* for 15 min. The supernatant was filtered through a 0.22-μm cellulose filter again and used for the quantitative analysis of amino acids or polyamines.

Concentrations of putrescine, cadaverine, spermidine, and spermine in the supernatant were determined using the LC-20A HPLC system (Shimadzu, Kyoto, Japan) coupled with an API 4000 Quantiva triple-quadrupole tandem mass spectrometer (SCIEX, Framingham, MA, USA) according to a previous study (79). HPLC separation was performed at 0.30 ml/min with the column compartment at 40°C. The stationary phase was an Inertsil ODS-3 column (2.1 by 100 mm; 3 μm), and the mobile phase was an isocratic mixture (20:80, A to B) of 5 mmol/liter CH_3_COONH_4_ in H_2_O (A) and 0.2% HCOOH in H_2_O (vol/vol) (B). Amino acids in the supernatant were determined by using the amino acid analyzer A300-advanced (membraPure, Berlin, Germany).

### Statistical information.

Results are expressed as means and standard errors (*n* = 3) as noted. Student's *t* test was used to calculate the *P* value as noted. *P* values of <0.05 and <0.01 were considered to indicate significant differences and highly significant differences, respectively.

### Data availability.

The genomes described in this study have been deposited in NCBI under GenBank accession number VUYS00000000 for “*Ca.* Nitrosocosmicus agrestis” and JAEPRP000000000 for heterotroph *Ensifer* sp. SSB1.

## Supplementary Material

Reviewer comments

## References

[1] Stahl DA, de la Torre JR. 2012. Physiology and diversity of ammonia-oxidizing archaea. Annu Rev Microbiol 66:83–101. doi:10.1146/annurev-micro-092611-150128. 22994489

[2] Kuypers MMM, Marchant HK, Kartal B. 2018. The microbial nitrogen-cycling network. Nat Rev Microbiol 16:263–276. doi:10.1038/nrmicro.2018.9. 29398704

[3] Stein LY. 2019. Insights into the physiology of ammonia-oxidizing microorganisms. Curr Opin Chem Biol 49:9–15. doi:10.1016/j.cbpa.2018.09.003. 30236860

[4] Leininger S, Urich T, Schloter M, Schwark L, Qi J, Nicol GW, Prosser JI, Schuster SC, Schleper C. 2006. Archaea predominate among ammonia-oxidizing prokaryotes in soils. Nature 442:806–809. doi:10.1038/nature04983. 16915287

[5] Liu L, Li S, Han J, Lin W, Luo J. 2019. A two-step strategy for the rapid enrichment of *Nitrosocosmicus*-like ammonia-oxidizing Thaumarchaea. Front Microbiol 10:875. doi:10.3389/fmicb.2019.00875. 31105671 PMC6491936

[6] Alves RJE, Kerou M, Zappe A, Bittner R, Abby SS, Schmidt HA, Pfeifer K, Schleper C. 2019. Ammonia oxidation by the arctic terrestrial thaumarchaeote *Candidatus* Nitrosocosmicus arcticus is stimulated by increasing temperatures. Front Microbiol 10:1571. doi:10.3389/fmicb.2019.01571. 31379764 PMC6657660

[7] Sauder LA, Albertsen M, Engel K, Schwarz J, Nielsen PH, Wagner M, Neufeld JD. 2017. Cultivation and characterization of *Candidatus* Nitrosocosmicus exaquare, an ammonia-oxidizing archaeon from a municipal wastewater treatment system. ISME J 11:1142–1157. doi:10.1038/ismej.2016.192. 28195581 PMC5398378

[8] Lehtovirta-Morley LE, Ross J, Hink L, Weber EB, Gubry-Rangin C, Thion C, Prosser JI, Nicol GW. 2016. Isolation of “*Candidatus* Nitrosocosmicus franklandus”, a novel ureolytic soil archaeal ammonia oxidiser with tolerance to high ammonia concentration. FEMS Microbiol Ecol 92:fiw057. doi:10.1093/femsec/fiw057. 26976843 PMC4830249

[9] Jung MY, Kim JG, Sinninghe Damsté JS, Rijpstra WIC, Madsen EL, Kim SJ, Hong H, Si OJ, Kerou M, Schleper C, Rhee SK. 2016. A hydrophobic ammonia-oxidizing archaeon of the Nitrosocosmicus clade isolated from coal tar-contaminated sediment. Environ Microbiol Rep 8:983–992. doi:10.1111/1758-2229.12477. 27700018

[10] Könneke M, Bernhard AE, de la Torre JR, Walker CB, Waterbury JB, Stahl DA. 2005. Isolation of an autotrophic ammonia-oxidizing marine archaeon. Nature 437:543–546. doi:10.1038/nature03911. 16177789

[11] Martens-Habbena W, Berube PM, Urakawa H, De La Torre JR, Stahl DA. 2009. Ammonia oxidation kinetics determine niche separation of nitrifying Archaea and Bacteria. Nature 461:976–979. doi:10.1038/nature08465. 19794413

[12] Dimitri Kits K, Sedlacek CJ, Lebedeva EV, Han P, Bulaev A, Pjevac P, Daebeler A, Romano S, Albertsen M, Stein LY, Daims H, Wagner M. 2017. Kinetic analysis of a complete nitrifier reveals an oligotrophic lifestyle. Nature 549:269–272. doi:10.1038/nature23679. 28847001 PMC5600814

[13] Berg C, Vandieken V, Thamdrup B, Jürgens K. 2015. Significance of archaeal nitrification in hypoxic waters of the Baltic Sea. ISME J 9:1319–1332. doi:10.1038/ismej.2014.218. 25423026 PMC4438320

[14] Trimmer M, Chronopoulou PM, Maanoja ST, Upstill-Goddard RC, Kitidis V, Purdy KJ. 2016. Nitrous oxide as a function of oxygen and archaeal gene abundance in the North Pacific. Nat Commun 7:13451. doi:10.1038/ncomms13451. 27905393 PMC5146275

[15] Sterngren AE, Hallin S, Bengtson P. 2015. Archaeal ammonia oxidizers dominate in numbers, but bacteria drive gross nitrification in *N*-amended grassland soil. Front Microbiol 6:1350. doi:10.3389/fmicb.2015.01350. 26648926 PMC4663241

[16] Egan G, Zhou X, Wang D, Jia Z, Crawley M, Fornara DA. 2018. Long-term effects of grazing, liming and nutrient fertilization on the nitrifying community of grassland soils. Soil Biol Biochem 118:97–102. doi:10.1016/j.soilbio.2017.12.005.

[17] Wang Q, Zhang LM, Shen JP, Du S, Han LL, He JZ. 2016. Nitrogen fertiliser-induced changes in N_2_O emissions are attributed more to ammonia-oxidising bacteria rather than archaea as revealed using 1-octyne and acetylene inhibitors in two arable soils. Biol Fertil Soils 52:1163–1171. doi:10.1007/s00374-016-1151-3.

[18] Hink L, Nicol GW, Prosser JI. 2017. Archaea produce lower yields of N_2_O than bacteria during aerobic ammonia oxidation in soil. Environ Microbiol 19:4829–4837. doi:10.1111/1462-2920.13282. 26971439

[19] Meinhardt KA, Stopnisek N, Pannu MW, Strand SE, Fransen SC, Casciotti KL, Stahl DA. 2018. Ammonia-oxidizing bacteria are the primary N_2_O producers in an ammonia-oxidizing archaea dominated alkaline agricultural soil. Environ Microbiol 20:2195–2206. doi:10.1111/1462-2920.14246. 29687586

[20] Duan P, Fan C, Zhang Q, Xiong Z. 2019. Overdose fertilization induced ammonia-oxidizing archaea producing nitrous oxide in intensive vegetable fields. Sci Total Environ 650:1787–1794. doi:10.1016/j.scitotenv.2018.09.341. 30278423

[21] Stopnišek N, Gubry-Rangin C, Höfferle Š, Nicol GW, Mandič-Mulec I, Prosser JI. 2010. Thaumarchaeal ammonia oxidation in an acidic forest peat soil is not influenced by ammonium amendment. Appl Environ Microbiol 76:7626–7634. doi:10.1128/AEM.00595-10. 20889787 PMC2976176

[22] Lu X, Bottomley PJ, Myrold DD. 2015. Contributions of ammonia-oxidizing archaea and bacteria to nitrification in Oregon forest soils. Soil Biol Biochem 85:54–62. doi:10.1016/j.soilbio.2015.02.034.

[23] Schauss K, Focks A, Leininger S, Kotzerke A, Heuer H, Thiele-Bruhn S, Sharma S, Wilke BM, Matthies M, Smalla K, Munch JC, Amelung W, Kaupenjohann M, Schloter M, Schleper C. 2009. Dynamics and functional relevance of ammonia-oxidizing archaea in two agricultural soils. Environ Microbiol 11:446–456. doi:10.1111/j.1462-2920.2008.01783.x. 19196275

[24] Koops H, Purkhold U, Pommerening-Röser A, Timmermann G, Wagner M. 2006. The lithoautotrophic ammonia-oxidizing bacteria, p 778–811. *In* Dworkin M, Falkow S, Rosenberg E, Schliefer K-H, Stackebrandt E (ed), The Prokaryotes, 3rd ed. Springer, New York, NY.

[25] Varghese NJ, Mukherjee S, Ivanova N, Konstantinidis KT, Mavrommatis K, Kyrpides NC, Pati A. 2015. Microbial species delineation using whole genome sequences. Nucleic Acids Res 43:6761–6771. doi:10.1093/nar/gkv657. 26150420 PMC4538840

[26] Luo C, Rodriguez-R LM, Konstantinidis KT. 2014. MyTaxa: an advanced taxonomic classifier for genomic and metagenomic sequences. Nucleic Acids Res 42:e73. doi:10.1093/nar/gku169. 24589583 PMC4005636

[27] Sauder LA, Engel K, Lo CC, Chain P, Neufeld JD. 2018. “*Candidatus* Nitrosotenuis aquarius,” an ammonia-oxidizing archaeon from a freshwater aquarium biofilter. Appl Environ Microbiol 84:e01430-18. doi:10.1128/AEM.01430-18. 29959256 PMC6146995

[28] Li Y, Ding K, Wen X, Zhang B, Shen B, Yang Y. 2016. A novel ammonia-oxidizing archaeon from wastewater treatment plant: its enrichment, physiological and genomic characteristics. Sci Rep 6:23747. doi:10.1038/srep23747. 27030530 PMC4814877

[29] Lebedeva EV, Hatzenpichler R, Pelletier E, Schuster N, Hauzmayer S, Bulaev A, Grigor'eva NV, Galushko A, Schmid M, Palatinszky M, Le Paslier D, Daims H, Wagner M. 2013. Enrichment and genome sequence of the group I.1a ammonia-oxidizing archaeon “*Ca.* Nitrosotenuis uzonensis” representing a clade globally distributed in thermal habitats. PLoS One 8:e80835. doi:10.1371/journal.pone.0080835. 24278328 PMC3835317

[30] Jung MY, Park SJ, Min D, Kim JS, Rijpstra WIC, Sinninghe Damste JS, Kim GJ, Madsen EL, Rhee SK. 2011. Enrichment and characterization of an autotrophic ammonia-oxidizing archaeon of mesophilic crenarchaeal group I.1a from an agricultural soil. Appl Environ Microbiol 77:8635–8647. doi:10.1128/AEM.05787-11. 22003023 PMC3233086

[31] Lehtovirta-Morley LE, Verhamme DT, Nicol GW, Prosser JI. 2013. Effect of nitrification inhibitors on the growth and activity of *Nitrosotalea devanaterra* in culture and soil. Soil Biol Biochem 62:129–133. doi:10.1016/j.soilbio.2013.01.020.

[32] Shen T, Stieglmeier M, Dai J, Urich T, Schleper C. 2013. Responses of the terrestrial ammonia-oxidizing archaeon *Ca.* Nitrososphaera viennensis and the ammonia-oxidizing bacterium *Nitrosospira multiformis* to nitrification inhibitors. FEMS Microbiol Lett 344:121–129. doi:10.1111/1574-6968.12164. 23617238

[33] Beeckman F, Motte H, Beeckman T. 2018. Nitrification in agricultural soils: impact, actors and mitigation. Curr Opin Biotechnol 50:166–173. doi:10.1016/j.copbio.2018.01.014. 29414056

[34] Kim JG, Jung MY, Park SJ, Rijpstra WIC, Sinninghe Damsté JS, Madsen EL, Min D, Kim JS, Kim GJ, Rhee SK. 2012. Cultivation of a highly enriched ammonia-oxidizing archaeon of thaumarchaeotal group I.1b from an agricultural soil. Environ Microbiol 14:1528–1543. doi:10.1111/j.1462-2920.2012.02740.x. 22515152

[35] Smith KS, Ferry JG. 2000. Prokaryotic carbonic anhydrases. FEMS Microbiol Rev 24:335–366. doi:10.1111/j.1574-6976.2000.tb00546.x. 10978542

[36] Ferry JG. 2010. The γ class of carbonic anhydrases. Biochim Biophys Acta 1804:374–381. doi:10.1016/j.bbapap.2009.08.026. 19747990 PMC2818130

[37] Kerou M, Offre P, Valledor L, Abby SS, Melcher M, Nagler M, Weckwerth W, Schleper C. 2016. Proteomics and comparative genomics of *Nitrososphaera viennensis* reveal the core genome and adaptations of archaeal ammonia oxidizers. Proc Natl Acad Sci U S A 113:E7937–E7946. doi:10.1073/pnas.1601212113. 27864514 PMC5150414

[38] Thoden JB, Huang X, Raushel FM, Holden HM. 2002. Carbamoyl-phosphate synthetase: creation of an escape route for ammonia. J Biol Chem 277:39722–39727. doi:10.1074/jbc.M206915200. 12130656

[39] Michael AJ. 2018. Polyamine function in archaea and bacteria. J Biol Chem 293:18693–18701. doi:10.1074/jbc.TM118.005670. 30254075 PMC6290158

[40] Pegg AE. 2013. Toxicity of polyamines and their metabolic products. Chem Res Toxicol 26:1782–1800. doi:10.1021/tx400316s. 24224555

[41] Dassler T, Maier T, Winterhalter C, Böck A. 2000. Identification of a major facilitator protein from *Escherichia coli* involved in efflux of metabolites of the cysteine pathway. Mol Microbiol 36:1101–1112. doi:10.1046/j.1365-2958.2000.01924.x. 10844694

[42] Zakataeva NP, Kutukova EA, Gronskiy SV, Troshin PV, Livshits VA, Aleshin VV. 2006. Export of metabolites by the proteins of the DMT and RhtB families and its possible role in intercellular communication. Microbiology 75:438–448. doi:10.1134/S0026261706040126. 17025177

[43] Igarashi K, Kashiwagi K. 2010. Characteristics of cellular polyamine transport in prokaryotes and eukaryotes. Plant Physiol Biochem 48:506–512. doi:10.1016/j.plaphy.2010.01.017. 20159658

[44] Tomitori H, Kashiwagi K, Asakawa T, Kakinuma Y, Michael AJ, Igarashi K. 2001. Multiple polyamine transport systems on the vacuolar membrane in yeast. Biochem J 353:681–688. doi:10.1042/0264-6021:3530681. 11171066 PMC1221615

[45] Michael AJ. 2016. Biosynthesis of polyamines and polyamine-containing molecules. Biochem J 473:2315–2329. doi:10.1042/BCJ20160185. 27470594

[46] He X, Ji G. 2020. Responses of AOA and AOB activity and DNA/cDNA community structure to allylthiourea exposure in the water level fluctuation zone soil. Environ Sci Pollut Res Int 27:15233–15244. doi:10.1007/s11356-020-07952-9. 32072408

[47] Wang M, Wang S, Long X, Zhuang L, Zhao X, Jia Z, Zhu G. 2019. High contribution of ammonia-oxidizing archaea (AOA) to ammonia oxidation related to a potential active AOA species in various arable land soils. J Soils Sediments 19:1077–1087. doi:10.1007/s11368-018-2108-y.

[48] Zhang MM, Alves RJE, Zhang DD, Han LL, He JZ, Zhang LM. 2017. Time-dependent shifts in populations and activity of bacterial and archaeal ammonia oxidizers in response to liming in acidic soils. Soil Biol Biochem 112:77–89. doi:10.1016/j.soilbio.2017.05.001.

[49] Hink L, Gubry-Rangin C, Nicol GW, Prosser JI. 2018. The consequences of niche and physiological differentiation of archaeal and bacterial ammonia oxidisers for nitrous oxide emissions. ISME J 12:1084–1093. doi:10.1038/s41396-017-0025-5. 29386627 PMC5864188

[50] Li X, Jayachandran S, Nguyen HHT, Chan MK. 2007. Structure of the *Nitrosomonas europaea* Rh protein. Proc Natl Acad Sci U S A 104:19279–19284. doi:10.1073/pnas.0709710104. 18040042 PMC2148281

[51] Lupo D, Li XD, Durand A, Tomizaki T, Cherif-Zahar B, Matassi G, Merrick M, Winkler FK. 2007. The 1.3-Å resolution structure of *Nitrosomonas europaea* Rh50 and mechanistic implications for NH_3_ transport by Rhesus family proteins. Proc Natl Acad Sci U S A 104:19303–19308. doi:10.1073/pnas.0706563104. 18032606 PMC2148285

[52] Ip YK, Chew SF. 2010. Ammonia production, excretion, toxicity, and defense in fish: a review. Front Physiol 1:134. doi:10.3389/fphys.2010.00134. 21423375 PMC3059970

[53] Tsuchiya H, Doki S, Takemoto M, Ikuta T, Higuchi T, Fukui K, Usuda Y, Tabuchi E, Nagatoishi S, Tsumoto K, Nishizawa T, Ito K, Dohmae N, Ishitani R, Nureki O. 2016. Structural basis for amino acid export by DMT superfamily transporter YddG. Nature 534:417–420. doi:10.1038/nature17991. 27281193

[54] Jack DL, Yang NM, Saier MH. 2001. The drug/metabolite transporter superfamily. Eur J Biochem 268:3620–3639. doi:10.1046/j.1432-1327.2001.02265.x. 11432728

[55] Bayer B, Hansman RL, Bittner MJ, Noriega-Ortega BE, Niggemann J, Dittmar T, Herndl GJ. 2019. Ammonia-oxidizing archaea release a suite of organic compounds potentially fueling prokaryotic heterotrophy in the ocean. Environ Microbiol 21:4062–4075. doi:10.1111/1462-2920.14755. 31336026 PMC6899801

[56] Bayer B, Pelikan C, Bittner MJ, Reinthaler T, Könneke M, Herndl GJ, Offre P. 2019. Proteomic response of three marine ammonia-oxidizing archaea to hydrogen peroxide and their metabolic interactions with a heterotrophic Alphaproteobacterium. mSystems 4:e00181-19. doi:10.1128/mSystems.00181-19. 31239395 PMC6593220

[57] Igarashi K, Kashiwagi K. 1996. Polyamine transport in Eseherichia coli. Amino Acids 10:83–97. doi:10.1007/BF00806095. 24178434

[58] Rudder S, Doohan F, Creevey CJ, Wendt T, Mullins E. 2014. Genome sequence of *Ensifer adhaerens* OV14 provides insights into its ability as a novel vector for the genetic transformation of plant genomes. BMC Genomics 15:268. doi:10.1186/1471-2164-15-268. 24708309 PMC4051167

[59] Williams LE, Baltrus DA, O’Donnell SD, Skelly TJ, Martin MO. 2017. Complete genome sequence of the predatory bacterium *Ensifer adhaerens* Casida A. Genome Announc 5:5–6. doi:10.1128/genomeA.01344-17. PMC5701487 29167262

[60] Krysenko S, Okoniewski N, Kulik A, Matthews A, Grimpo J, Wohlleben W, Bera A. 2017. Gamma-glutamylpolyamine synthetase GlnA3 is involved in the first step of polyamine degradation pathway in *Streptomyces coelicolor* M145. Front Microbiol 8:726. doi:10.3389/fmicb.2017.00726. 28487688 PMC5403932

[61] Schneider BL, Reitzer L. 2012. Pathway and enzyme redundancy in putrescine catabolism in *Escherichia coli*. J Bacteriol 194:4080–4088. doi:10.1128/JB.05063-11. 22636776 PMC3416515

[62] Alves RJE, Minh BQ, Urich T, Von Haeseler A, Schleper C. 2018. Unifying the global phylogeny and environmental distribution of ammonia-oxidising archaea based on *amoA* genes. Nat Commun 9:1517. doi:10.1038/s41467-018-03861-1. 29666365 PMC5904100

[63] ISO/TS 14256-1. 2003. Soil quality—determination of nitrate, nitrite and ammonium in field-moist soils by extraction with potassium chloride solution. Part 1: Manual method. Iso/Ts 14256-1. International Organisation for Standardisation, Geneva, Switzerland.

[64] Emerson K, Russo RC, Lund RE, Thurston RV. 1975. Aqueous ammonia equilibrium calculations: effect of pH and temperature. J Fish Res Bd Can 32:2379–2383. doi:10.1139/f75-274.

[65] Bolger AM, Lohse M, Usadel B. 2014. Trimmomatic: a flexible trimmer for Illumina sequence data. Bioinformatics 30:2114–2120. doi:10.1093/bioinformatics/btu170. 24695404 PMC4103590

[66] Nurk S, Meleshko D, Korobeynikov A, Pevzner PA. 2017. MetaSPAdes: a new versatile metagenomic assembler. Genome Res 27:824–834. doi:10.1101/gr.213959.116. 28298430 PMC5411777

[67] Luo R, Liu B, Xie Y, Li Z, Huang W, Yuan J, He G, Chen Y, Pan Q, Liu Y, Tang J, Wu G, Zhang H, Shi Y, Liu Y, Yu C, Wang B, Lu Y, Han C, Cheung DW, Yiu SM, Peng S, Xiaoqian Z, Liu G, Liao X, Li Y, Yang H, Wang J, Lam TW, Wang J. 2012. SOAPdenovo2: an empirically improved memory-efficient short-read de novo assembler. Gigascience 1:18. doi:10.1186/2047-217X-1-18. 23587118 PMC3626529

[68] Wu YW, Simmons BA, Singer SW. 2016. MaxBin 2.0: an automated binning algorithm to recover genomes from multiple metagenomic datasets. Bioinformatics 32:605–607. doi:10.1093/bioinformatics/btv638. 26515820

[69] Wu D, Jospin G, Eisen JA. 2013. Systematic identification of gene families for use as “markers” for phylogenetic and phylogeny-driven ecological studies of bacteria and archaea and their major subgroups. PLoS One 8:e77033. doi:10.1371/journal.pone.0077033. 24146954 PMC3798382

[70] Parks DH, Imelfort M, Skennerton CT, Hugenholtz P, Tyson GW. 2015. CheckM: assessing the quality of microbial genomes recovered from isolates, single cells, and metagenomes. Genome Res 25:1043–1055. doi:10.1101/gr.186072.114. 25977477 PMC4484387

[71] Chen IMA, Chu K, Palaniappan K, Pillay M, Ratner A, Huang J, Huntemann M, Varghese N, White JR, Seshadri R, Smirnova T, Kirton E, Jungbluth SP, Woyke T, Eloe-Fadrosh EA, Ivanova NN, Kyrpides NC. 2019. IMG/M v.5.0: an integrated data management and comparative analysis system for microbial genomes and microbiomes. Nucleic Acids Res 47:D666–D677. doi:10.1093/nar/gky901. 30289528 PMC6323987

[72] Vallenet D, Calteau A, Dubois M, Amours P, Bazin A, Beuvin M, Burlot L, Bussell X, Fouteau S, Gautreau G, Lajus A, Langlois J, Planel R, Roche D, Rollin J, Rouy Z, Sabatet V, Médigue C. 2020. MicroScope: an integrated platform for the annotation and exploration of microbial gene functions through genomic, pangenomic and metabolic comparative analysis. Nucleic Acids Res 48:D579–D589. doi:10.1093/nar/gkz926. 31647104 PMC7145621

[73] Moriya Y, Itoh M, Okuda S, Yoshizawa AC, Kanehisa M. 2007. KAAS: an automatic genome annotation and pathway reconstruction server. Nucleic Acids Res 35:W182–W185. doi:10.1093/nar/gkm321. 17526522 PMC1933193

[74] Saier MH, Reddy VS, Tamang DG, Västermark Å. 2014. The transporter classification database. Nucleic Acids Res 42:251–258. doi:10.1093/nar/gkt1097. PMC3964967 24225317

[75] Zhang H, Yohe T, Huang L, Entwistle S, Wu P, Yang Z, Busk PK, Xu Y, Yin Y. 2018. DbCAN2: a meta server for automated carbohydrate-active enzyme annotation. Nucleic Acids Res 46:W95–W101. doi:10.1093/nar/gky418. 29771380 PMC6031026

[76] Richter M, Rosselló-Móra R, Oliver Glöckner F, Peplies J. 2016. JSpeciesWS: a web server for prokaryotic species circumscription based on pairwise genome comparison. Bioinformatics 32:929–931. doi:10.1093/bioinformatics/btv681. 26576653 PMC5939971

[77] Kalyaanamoorthy S, Minh BQ, Wong TKF, Von Haeseler A, Jermiin LS. 2017. ModelFinder: fast model selection for accurate phylogenetic estimates. Nat Methods 14:587–589. doi:10.1038/nmeth.4285. 28481363 PMC5453245

[78] Nguyen LT, Schmidt HA, Von Haeseler A, Minh BQ. 2015. IQ-TREE: a fast and effective stochastic algorithm for estimating maximum-likelihood phylogenies. Mol Biol Evol 32:268–274. doi:10.1093/molbev/msu300. 25371430 PMC4271533

[79] Gosetti F, Mazzucco E, Gennaro MC, Marengo E. 2013. Simultaneous determination of sixteen underivatized biogenic amines in human urine by HPLC-MS/MS. Anal Bioanal Chem 405:907–916. doi:10.1007/s00216-012-6269-z. 22842827

[80] Park BJ, Park SJ, Yoon DN, Schouten S, Sinninghe Damsté JS, Rhee SK. 2010. Cultivation of autotrophic ammonia-oxidizing archaea from marine sediments in coculture with sulfur-oxidizing bacteria. Appl Environ Microbiol 76:7575–7587. doi:10.1128/AEM.01478-10. 20870784 PMC2976178

[81] Jung MY, Park SJ, Kim SJ, Kim JG, Sinninghe Damsté JS, Jeon CO, Rhee SK. 2014. A mesophilic, autotrophic, ammonia-oxidizing archaeon of thaumarchaeal group I.1a cultivated from a deep oligotrophic soil horizon. Appl Environ Microbiol 80:3645–3655. doi:10.1128/AEM.03730-13. 24705324 PMC4054128

[82] Qin W, Heal KR, Ramdasi R, Kobelt JN, Martens-Habbena W, Bertagnolli AD, Amin SA, Walker CB, Urakawa H, Könneke M, Devol AH, Moffett JW, Armbrust EV, Jensen GJ, Ingalls AE, Stahl DA. 2017. *Nitrosopumilus maritimus* gen. nov., sp. nov., *Nitrosopumilus cobalaminigenes* sp. nov., *Nitrosopumilus oxyclinae* sp. nov., and *Nitrosopumilus ureiphilus* sp. nov., four marine ammonia-oxidizing archaea of the phylum *Thaumarchaeota*. Int J Syst Evol Microbiol 67:5067–5079. doi:10.1099/ijsem.0.002416. 29034851

[83] Lehtovirta-Morley LE, Stoecker K, Vilcinskas A, Prosser JI, Nicol GW. 2011. Cultivation of an obligate acidophilic ammonia oxidizer from a nitrifying acid soil. Proc Natl Acad Sci U S A 108:15892–15897. doi:10.1073/pnas.1107196108. 21896746 PMC3179093

[84] Hatzenpichler R, Lebedeva EV, Spieck E, Stoecker K, Richter A, Daims H, Wagner M. 2008. A moderately thermophilic ammonia-oxidizing crenarchaeote from a hot spring. Proc Natl Acad Sci U S A 105:2134–2139. doi:10.1073/pnas.0708857105. 18250313 PMC2538889

[85] Stieglmeier M, Klingl A, Alves RJE, Rittmann SKMR, Melcher M, Leisch N, Schleper C. 2014. *Nitrososphaera viennensis* gen. nov., sp. nov., an aerobic and mesophilic, ammonia-oxidizing archaeon from soil and a member of the archaeal phylum *Thaumarchaeota*. Int J Syst Evol Microbiol 64:2738–2752. doi:10.1099/ijs.0.063172-0. 24907263 PMC4129164

[86] Chen H, Yue Y, Jin W, Zhou X, Wang Q, Gao S, Hong Jun Xie G, Du S, Tu R, Han S, Guo K. 2017. Enrichment and characteristics of ammonia-oxidizing archaea in wastewater treatment process. Chem Eng J 323:465–472. doi:10.1016/j.cej.2017.04.130.

