# Supplementary figures and images for: Production and Excretion of Polyamines To Tolerate High Ammonia, a Case Study on Soil Ammonia-Oxidizing Archaeon “Candidatus Nitrosocosmicus agrestis”
Source: mSystems. 2021 Feb 16;6(1):e01003-20. doi: 10.1128/mSystems.01003-20 (PMC8573960; doi:10.1128/mSystems.01003-20)

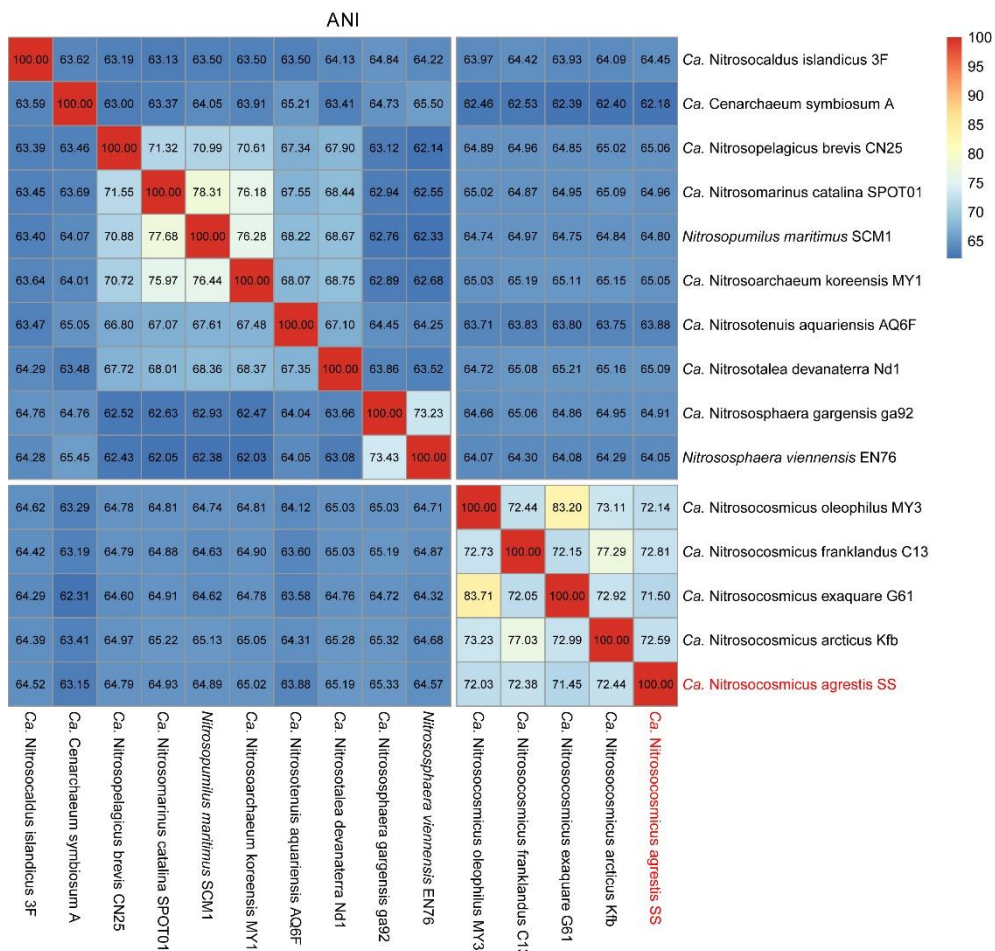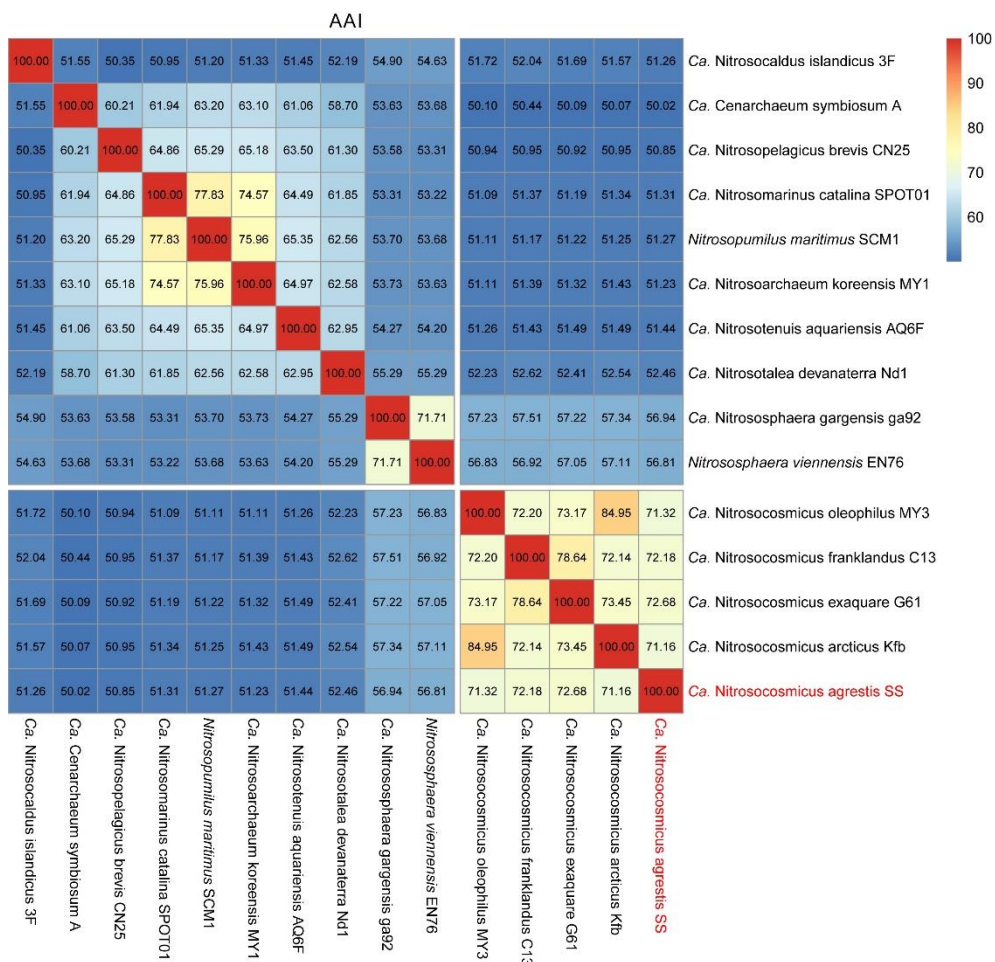

Supplement: FIG S1 [file msystems.01003-20-sf001.pdf]

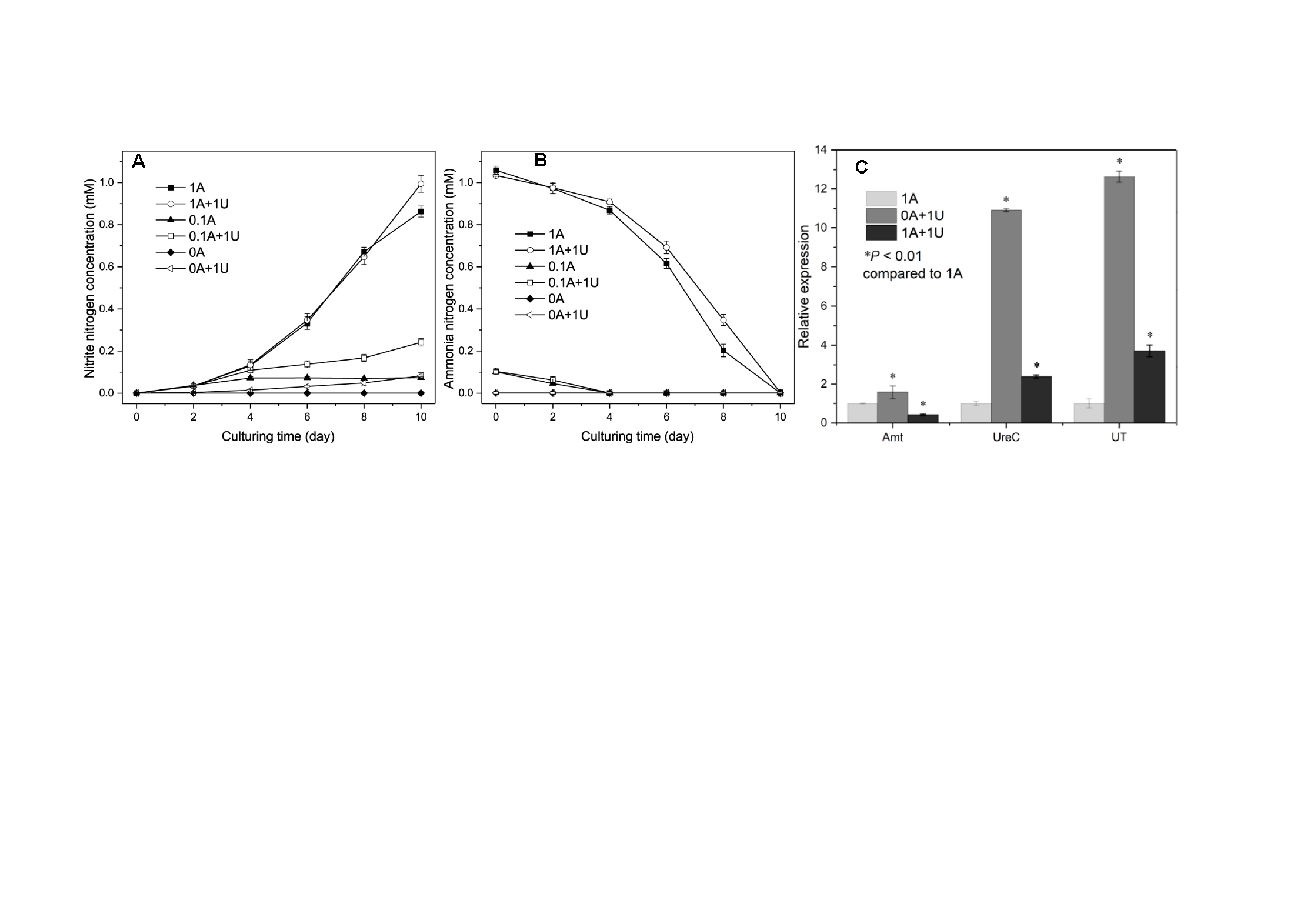

Supplement: FIG S2 [file msystems.01003-20-sf002.tif]

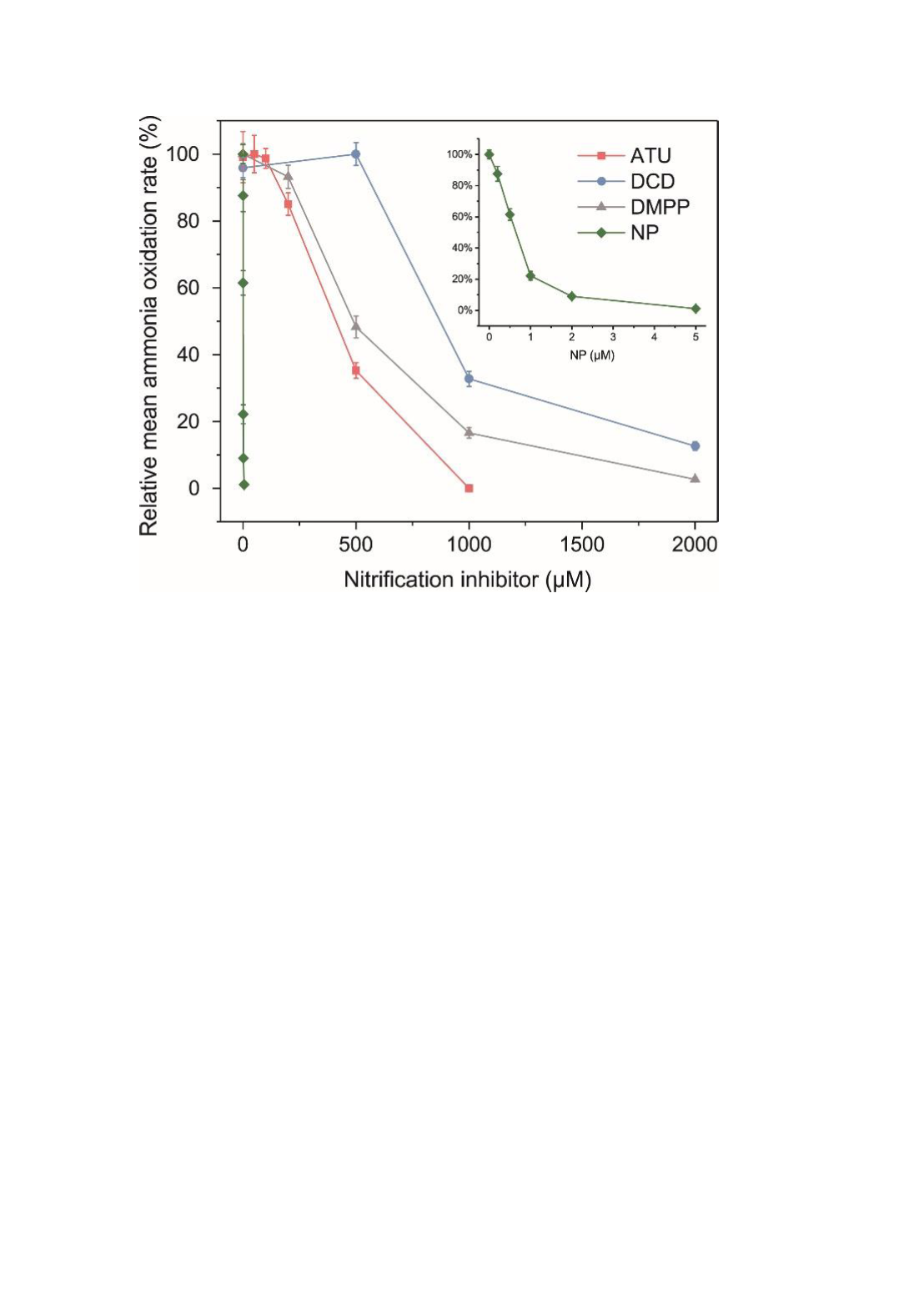

Supplement: FIG S3 [file msystems.01003-20-sf003.tif]

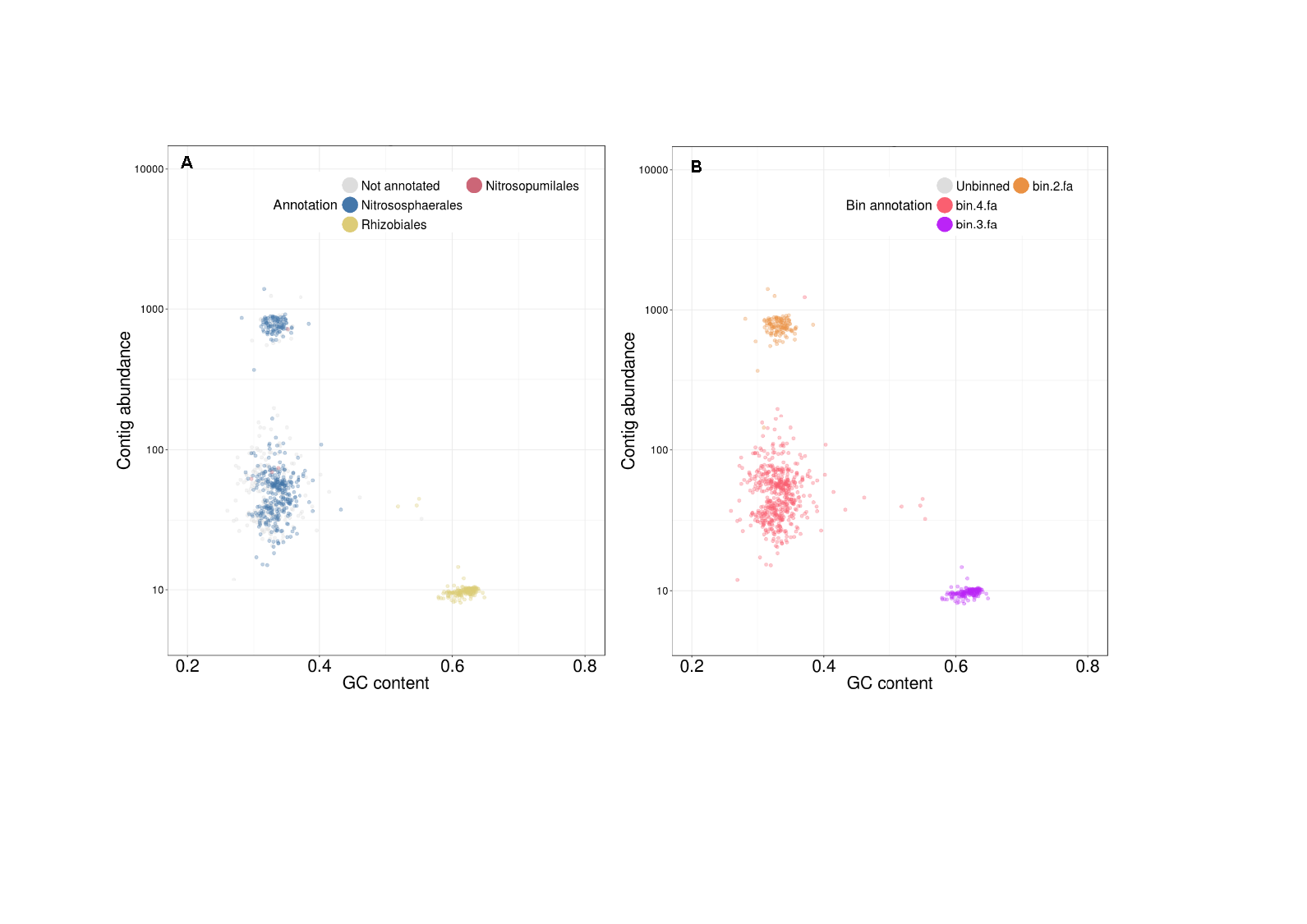

Supplement: FIG S4 [file msystems.01003-20-sf004.tif]

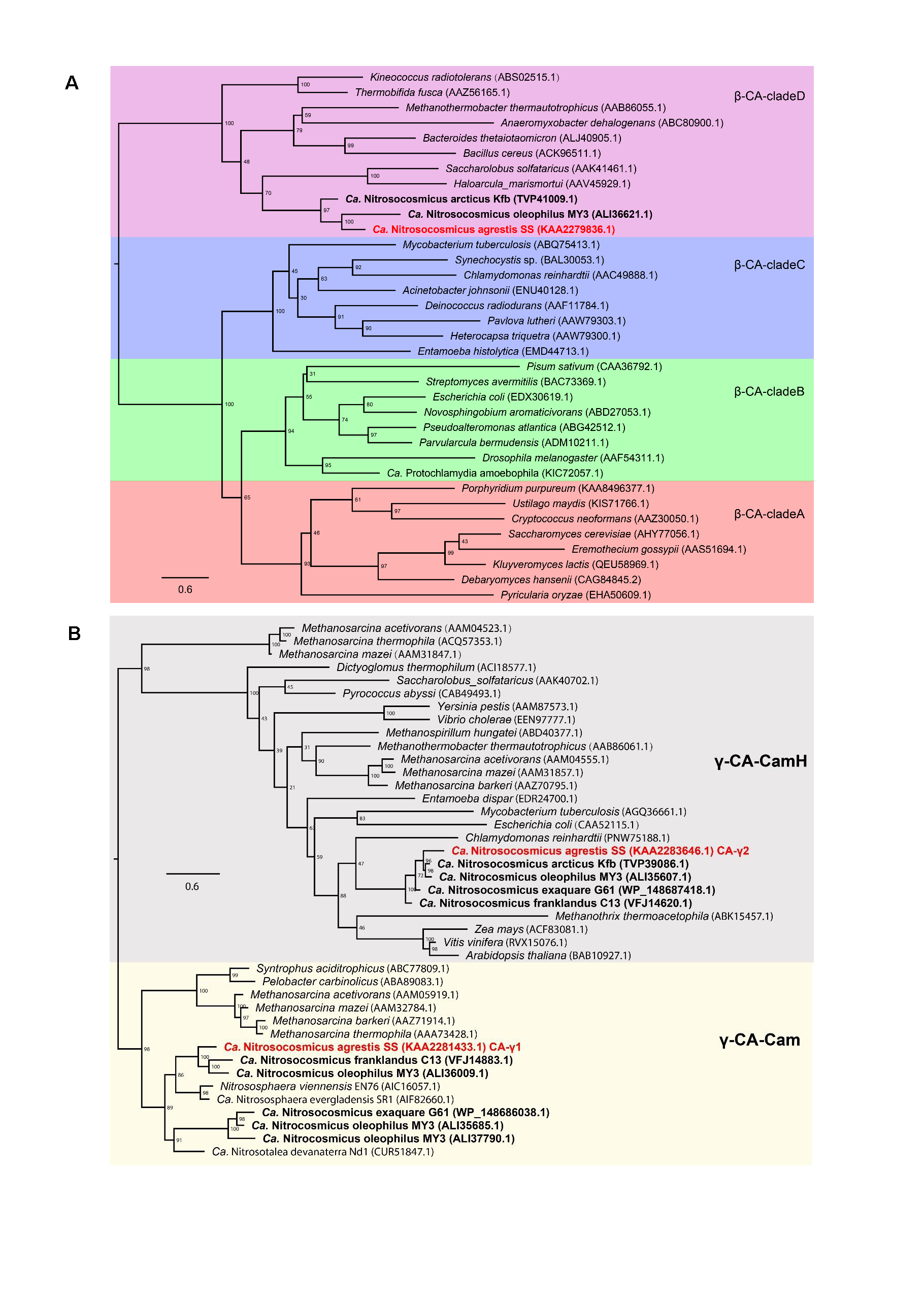

Supplement: FIG S5 [file msystems.01003-20-sf005.tif]

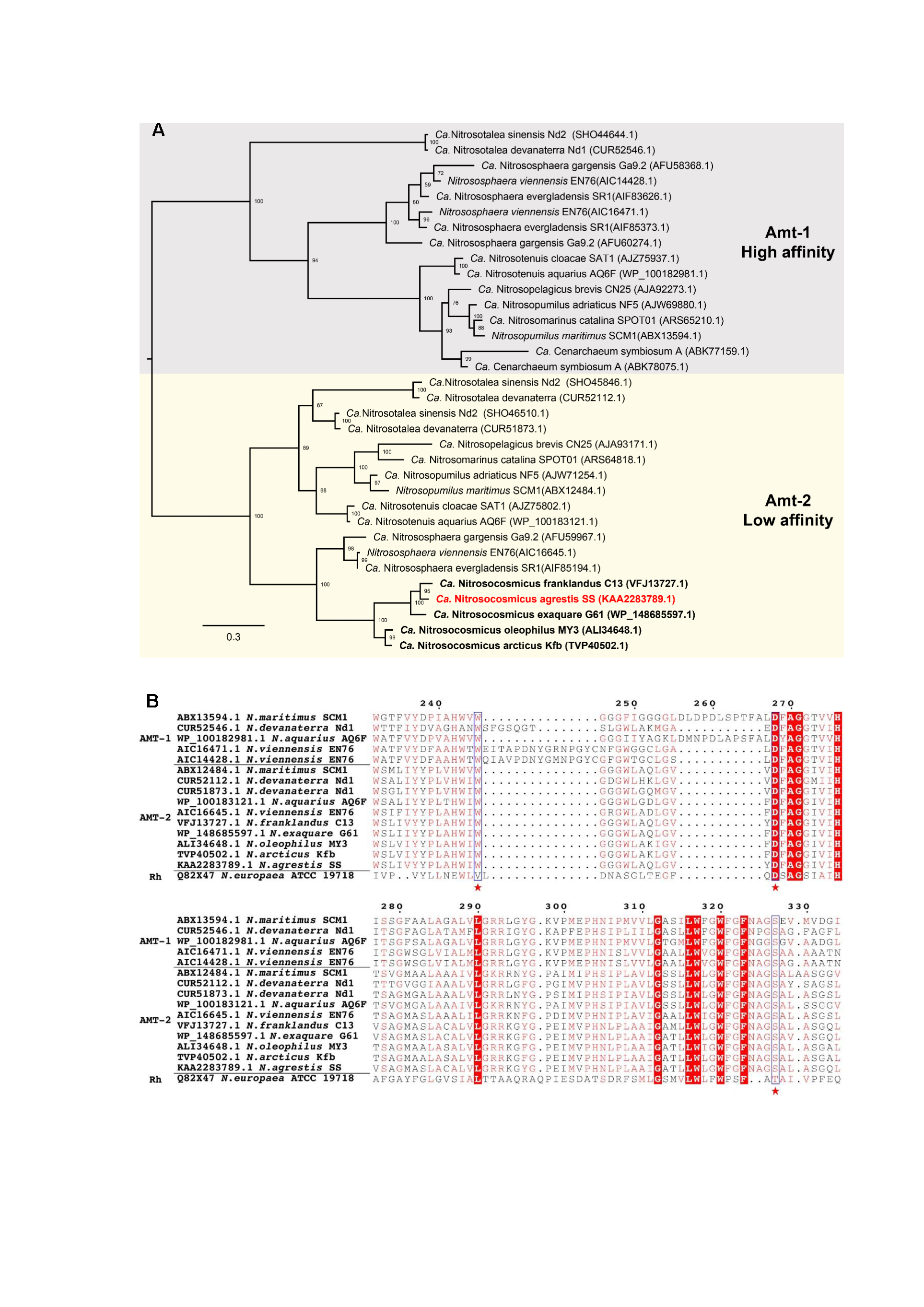

Supplement: FIG S6 [file msystems.01003-20-sf006.tif]

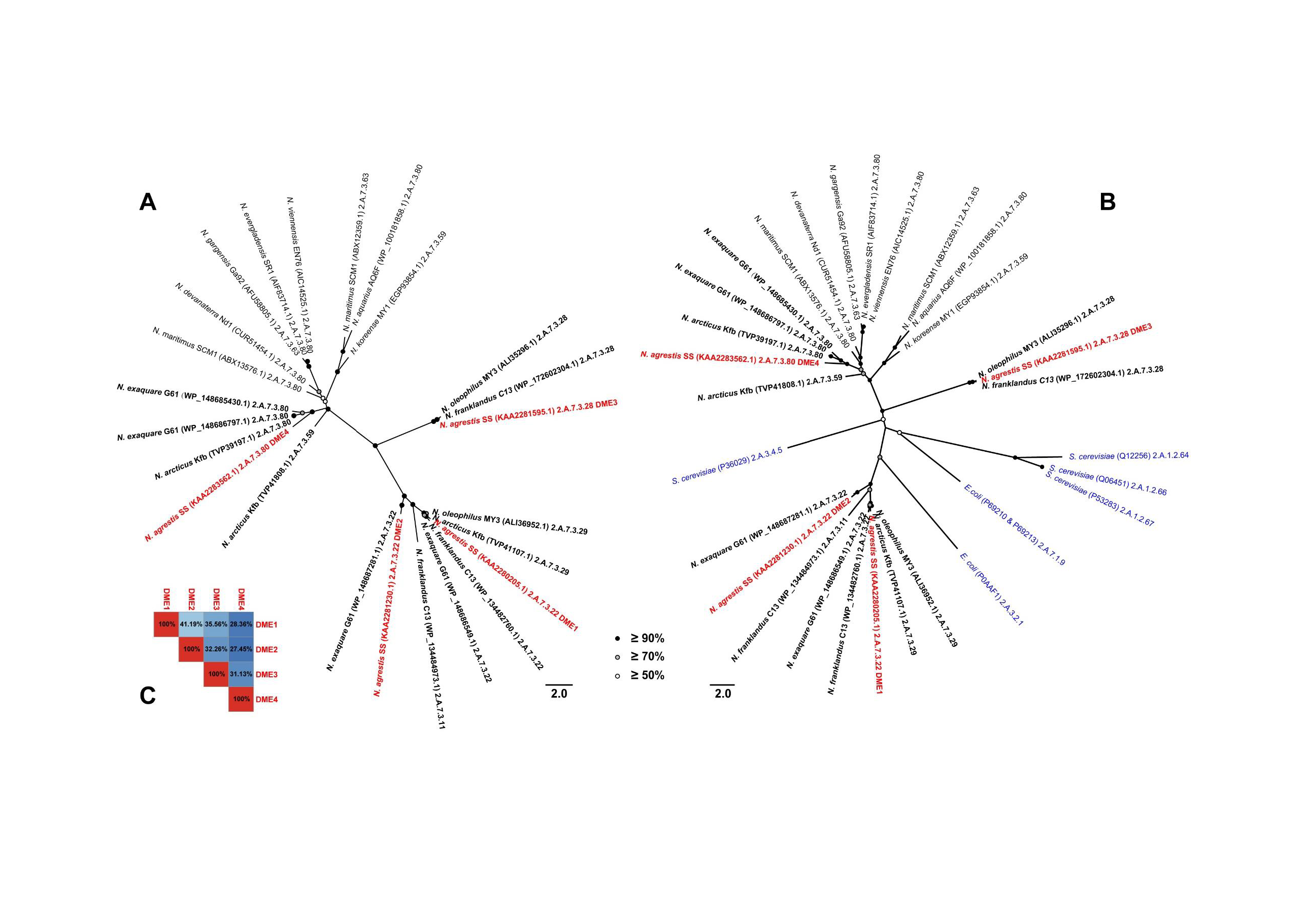

Supplement: FIG S7 [file msystems.01003-20-sf007.tif]
